# Associations of cardiovascular–kidney–metabolic syndrome stages with premature mortality and the role of social determinants of health

**DOI:** 10.1016/j.jnha.2025.100504

**Published:** 2025-02-13

**Authors:** Ruixin Zhu, Ran Wang, Jingjing He, Langrun Wang, Huiyu Chen, Yifan Wang, Peng An, Keji Li, Fazheng Ren, Weili Xu, J. Alfredo Martinez, Anne Raben, Jie Guo

**Affiliations:** a Key Laboratory of Precision Nutrition and Food Quality, Department of Nutrition and Health, China Agricultural University, Beijing, China; b Department of Nutrition and Food Hygiene, School of Public Health, Peking University, Beijing, China; c Department of Neurobiology, Care Sciences & Society, Karolinska Institutet, Solna, Sweden; d Centro de Investigacion Biomedica en Red Area de Fisiologia de la Obesidad y la Nutricion (CIBEROBN), Madrid, Spain; e Precision Nutrition and Cardiometabolic Health Program, IMDEA-Food Institute (Madrid Institute for Advanced Studies), CEI UAM + CSIC, Madrid, Spain; f Department of Medicine and Endocrinology, University of Valladolid, Valladolid, Spain; g Department of Nutrition, Exercise and Sports, Faculty of Science, University of Copenhagen, Copenhagen, Denmark; h Department for Clinical and Translational Research, Copenhagen University Hospital — Steno Diabetes Center Copenhagen, Herlev, Denmark

**Keywords:** Cardiovascular–kidney–metabolic health, Cardiovascular disease, Diabetes, Obesity, Health disparities

## Abstract

**Objectives:**

The American Heart Association defined cardiovascular–kidney–metabolic (CKM) syndrome as a novel multi-stage disorder. We examined the associations of CKM stages with premature mortality and the role of social determinants of health (SDOHs).

**Design:**

A prospective cohort study.

**Setting and participants:**

A nationally representative sample of US adults from NHANES 1999–2018.

**Measurements:**

CKM included 5 stages (stages 0–4), reflecting progressive pathophysiology. Premature mortality (deaths before 75 years) were ascertained via linkage to the National Death Index with follow-up until 2019. Cox proportional-hazards models adjusted for age, sex, race/ethnicity, medical history, and other confounding factors were used to calculate the hazard ratios (HR) and 95% CIs for CKM-mortality associations.

**Results:**

Among 27,909 participants (mean age 49.7 years, 49.0% females), 1762 premature deaths occurred over a median follow-up of 8.3 years. Compared with stage 0, the adjusted HRs for all-cause premature mortality at CKM stages 1–4 were 0.88 (95% CI 0.66–1.17), 1.31 (0.99–1.73), 1.94 (1.31–2.87), and 2.19 (1.61–2.98), respectively. For CVD premature mortality, the adjusted HRs for CKM stages 1–4 were 1.12 (0.46–2.72), 1.74 (0.71–4.28), 3.93 (1.53–10.12), and 6.48 (2.95–14.20), respectively. Among adults at CKM stages 3–4, unfavorable SDOHs, particularly not living with a partner, low family income, lack of private health insurance, unemployment, or ≥2 cumulative SDOHs (4.16, 95% CI 3.35–5.18) were associated with increased all-cause premature mortality. Among those at CKM stages 0–2, unfavorable SDOHs were also related to increased premature mortality.

**Conclusion:**

CKM stages 3–4, but not stages 1–2, were associated with increased risks of premature mortality compared with stage 0. The risks were increased by unfavorable SDOHs across CKM stages.

## Highlights

• Cardiovascular–kidney–metabolic (CKM) stages 3–4, but not stages 1–2, had higher premature mortality.
• Unfavorable social determinants of health were related to higher premature mortality across CKM stages.
• There were sex and age differences in the associations of CKM stages with premature mortality.

## 1 Introduction

Cardiovascular–kidney–metabolic (CKM) syndrome is a novel multi-system disorder defined by the American Heart Association (AHA) [1]. CKM syndrome reflects the complex interactions among obesity, diabetes, chronic kidney disease (CKD), and cardiovascular disease (CVD) and is classified into 5 stages based on the presence of CKM risk factors, ranging from 0 (no risk factors) to 4 (established CVD) [1]. The CKM staging system highlights the importance of co-management of comorbidity to maximize health care benefits, which has great clinical practical value. Evidence shows that there is high prevalence of poor CKM health in the US [2,3] and a high incidence of CKM syndrome in China [4]. Between 2011 and 2020, around 90% of US adults met the criteria for CKM syndrome and 15% met the criteria for stages 3 or 4 (advanced stages) [2].

Premature mortality, unconditional probability of deaths before the age of 70 or 75 years, is a widely used metric of population health and compared with natural death, premature mortality is more important to public health policy making, as it captures some dimension of an unnecessary or avoidable burden of mortality, life loss, and economic loss [5,6]. County-level data showed that in the US, CVD premature mortality rates related to CKM syndrome were high with wide variation across states [7]. Individual-level data showed that compared with CKM stage 0, stages 1–4 were associated with increased risks of all-cause mortality [8]. Nonetheless, the associations of CKM stages with all-cause and CVD premature mortality still remain unclear. Identifying the CKM stage at which the risk of premature mortality significantly increased and determining the stages that warrant more attention could aid in developing effective screening and prevention strategies.

Social determinants of health (SDOHs) are critical indicators of health equity, encompassing various non-medical factors that influence health, such as economic stability, education, food security, community and social context, and healthcare system [9,10]. Upstream SDOHs interact across these non-medical factors, leading to cumulative downstream effects on health [9]. Given their impact on health outcomes, SDOHs are considered as important as lifestyle factors [11]. Recent studies have shown that cumulative social disadvantage was associated with higher odds of CKM syndrome [12,13]. Our previous study found that advanced CKM stages 3–4 were highly prevalent in US adults with unfavorable SDOHs [3]. Furthermore, SDOHs were found to be associated with differences in morbidity and mortality related to the components of CKM syndrome, such as obesity, type 2 diabetes, CVD, and CKD [1,10,[14], [15], [16], [17]]. County-level data suggested that there were considerable geographical differences in the magnitude of the association of social and environmental determinants of health with CKM-related mortality across the US [18]. However, there are no individual-level data investigating the role of SDOHs in associations of specific CKM stages with all-cause and CVD premature mortality. Understanding the role of SDOHs may help development personalized and effective prevention and treatment strategies.

The main aim of this prospective cohort study was to examine the associations of CKM stages with all-cause and CVD premature mortality among US adults. Additionally, we examined the role of SDOHs in these associations.

## 2 Methods

### 2.1 Study design and participants

The National Health and Nutrition Examination Survey (NHANES) is a series of cross-sectional surveys with complex sampling to access the health and nutritional status in the non-institutionalized US population of all ages [19]. The participants were followed up until death. In the current prospective cohort study, participants’ data from 1999–2000 to 2017–2018 NHANES cycles were used. Deaths were identified by linking NHANES participants to the National Death Index (NDI).

Of a total of 101,316 participants, we excluded those who were pregnant or lactating. In addition, since the definition of CKM stages partially relied on the AHA Predicting Risk of CVD Events (PREVENT) equation (base model) [20], which is only valid for individuals aged 30–79 years and excludes those with extreme CVD risk factor values, we excluded participants younger than 30 or older than 79 years, as well as those with missing or extreme values (detailed information in eFig. 1). After further excluding 59 participants without eligible death information, we finally included 27,909 participants aged 30–74 years. The Institutional Review Board for the National Center for Health Statistics (now referred to as the Ethics Review Board) approved the NHANES protocol and allowed the data files to be posted on their website for public use. All participants provided written informed consent before data collection. This report followed the Strengthening the Reporting of Observational Studies in Epidemiology (STROBE) reporting guidelines for cohort studies.

### 2.2 Assessment of CKM stages

We classified CKM syndrome into 5 stages (i.e., stages 0–4), according to the criteria from both AHA and Aggarwal et al. [1,2] Detailed CKM stage definitions, adapted to data available in NHANES, have been previous described [19] and are outlined in eMethods and eTable 1. Briefly, stage 0 identified participants with a normal BMI and normal waist circumference who did not meet criteria for the other stages; stage 1 identified participants with an elevated BMI, an elevated waist circumference, or prediabetes; stage 2 identified participants with metabolic risk factors or moderate-to-high-risk CKD according to the Kidney Disease Improving Global Outcomes (KDIGO) criteria [21]; stage 3 identified participants with very-high-risk KDIGO CKD or a high-predicted 10-year CVD risk estimated using the PREVENT equations; stage 4 identified participants with self-reported CVD.

### 2.3 Ascertainment of outcomes

Outcomes included all-cause and CVD premature mortality. Deaths were identified by linking NHANES participants to the NDI, with follow-up until December 31, 2019. All-cause premature mortality was defined as deaths occurring before the age of 75, roughly corresponding to the average age of death in the US during the study period [22]. Causes of deaths were classified using the International Statistical Classification of Diseases Tenth Revision (ICD-10) codes and codes I00–109, I11, I13, I20–I51, and I60–I69 indicated deaths from CVD, I00–109, I11, I13, and I20–I51 indicated deaths from heart diseases, and I60–I69 indicated deaths from cerebrovascular diseases. In sensitivity analyses, premature mortality was defined as deaths occurring before 65, 70, and 80 years of age, according to the age of retirement in many countries and the World Health Organization criteria.

### 2.4 Assessment of covariates

Information on demographic factors (age, sex, and race/ethnicity), 8 SDOHs (i.e., education, marital status, family income-to-poverty, food security, health insurance, employment status, home ownership, and health-care access), and lifestyle factors (i.e., smoking status, alcohol consumption, and physical activity) were collected during household interview in each NHANES cycle using standard questionnaires. Race/ethnicity was self-identified by participants, including Mexican American, non-Hispanic Black, non-Hispanic White, other Hispanic, and other races (e.g., multi-racial). In the current analysis, other Hispanic and other races were combined due to their limited sample size. SDOHs were dichotomized based on conventional cut-points [10]. Cumulative unfavorable SDOHs were calculated by summing the 8 dichotomized SDOHs, assigning a value of 1 for each unfavorable level and 0 for each favorable level. These cumulative scores were further dichotomized as ≥2 versus <2 unfavorable SDOHs based on the median level. Detailed definitions of covariates are provided in eMethods.

### 2.5 Statistical analysis

Data were analyzed from March 2024 to September 2024. To account for the complex sampling design of NHANES, 20-year mobile examination center weights from 10 survey cycles were appropriately calculated and incorporated into all analyses. Differences in baseline characteristics across 5 CKM stages as well as between CKM stages 3–4 (advanced stages) and stages 0–2 (non-advanced stages) were assessed using survey-weighted Pearson’s chi-squared test for categorical variables and survey-weighted linear regression models for continuous variables.

Survey-weighted Cox proportional hazards models were performed to estimate hazard ratios (HRs) and 95% confidence intervals (CIs) for the associations between CKM stages and premature mortality from all causes or CVD or specific CVD (i.e., heart and cerebrovascular diseases). Proportional hazards assumptions were checked using Schoenfeld residuals, resulting in no violations. Follow-up time was defined as the time from the initial date of the NHANES survey cycle until the date of death, age of 75 years, or December 31, 2019, whichever occurred first. In the Cox models, we adjusted for age, sex, and race/ethnicity (model 1), followed by additional adjustments for education, marital status, family income-to-poverty ratio, food security, type of health insurance, employment, home ownership, regular health-care access, alcohol consumption, smoking status, physical activity, and medical history of cancer at baseline (model 2). We further examined the joint associations of CKM stages with age, sex, race/ethnicity, and all 8 SDOHs with premature mortality. Multiplicative interactions between CKM stages and these factors were assessed by including their cross-product term in the model. This study was designed to generate rather than test hypotheses and adjustments for multiple comparisons were therefore not applied. The results should be interpreted as exploratory because of the potential for type I errors.

In the sensitivity analyses, we performed Fine and Gray cox regression to estimate the associations of CKM stages and combined CKM stages and SDOHs with CVD premature mortality while accounting for competing events (i.e., deaths from cancer and other causes). In addition, to minimize reverse causality, we excluded person years during the first 2 years of follow-up. We conducted subgroup analyses by 8 SDOHs to explore the associations between CKM stages and premature mortality. Additionally, subgroup analyses by CKM stages were performed to explore the associations between these variables and premature mortality. Sex- and age-stratified analyses were performed to explore the associations between the combination of CKM stages with all 8 SDOHs and premature mortality.

All statistical analyses were performed using SAS software (version 9.4, SAS Institute, Cary, NC) with SURVEYFREQ, SURVEYMEANS, SURVEYLOGISTIC, SURVEYREG, or SURVEYPHREG procedure. Missing data were removed by the statistical software automatically. All *P* values were two-sided, and statistical significance was defined as *P* < 0.05.

## 3 Results

### 3.1 Participants’ characteristics

Of 27,909 participants included in this study, the weighted mean age (SE) was 49.7 (0.1) years. Among them, 13,614 were females (weighted percentage 49.0%), 14,295 were males (51.0%), 5244 were Mexican American (7.7%), 5373 were Non-Hispanic Black (9.4%), 11,998 were Non-Hispanic White (70.5%), and 5294 were others (12.4%). Participants with CKM stages 3–4 (advanced stages) were more likely to be older, male, smokers, non-drinkers, physically inactive, and users of anti-hypertensive and lipid-lowering medications and more likely to have unfavorable SDOHs (Table 1).

**Table 1:** Baseline characteristics of participants according to cardiovascular–kidney–metabolic syndrome stages.

Characteristic	Cardiovascular–kidney–metabolic syndrome stage								
	Participants, no, (%)^a^								
	0	1	2	3	4	*P* values^b^	Stages 0–2 (Non-advanced stages)	Stages 3–4 (Advanced stages)	*P* values^b^
No. of participants	3197	7904	12,962	1344	2502		24,063	3846	
Age, mean (SE), years	43.7 (0.3)	45.0 (0.2)	51.9 (0.2)	68.0 (0.3)	59.8 (0.3)		48.2 (0.1)	62.1 (0.2)	
Age group (n = 27,909)						<0.001			<0.001
30–59 years	2853 (91.5)	6829 (88.8)	8276 (72.3)	90 (8.9)	863 (42.4)		17,958 (81.0)	953 (33.0)	
60–74 years	344 (8.5)	1075 (11.2)	4686 (27.7)	1254 (91.1)	1639 (57.6)		6105 (19.0)	2893 (67.0)	
Sex (n = 27,909)						<0.001			<0.001
Male	1387 (40.1)	3918 (50.5)	6655 (52.7)	869 (62.9)	1466 (58.8)		11,960 (50.0)	2335 (60.0)	
Female	1810 (59.9)	3986 (49.5)	6307 (47.3)	475 (37.1)	1036 (41.2)		12,103 (50.0)	1511 (40.0)	
Race and ethnicity (n = 27,909)						<0.001			<0.001
Mexican American	377 (4.6)	1713 (10.0)	2516 (7.6)	276 (7.0)	362 (4.7)		4606 (8.0)	638 (5.3)	
Non-Hispanic Black	401 (5.9)	1406 (9.2)	2652 (10.1)	349 (12.8)	565 (11.0)		4459 (9.1)	914 (11.5)	
Non-Hispanic White	1692 (76.2)	3307 (68.2)	5299 (69.8)	504 (68.7)	1196 (73.3)		10,298 (70.3)	1700 (72.0)	
Other^c^	727 (13.2)	1478 (12.5)	2495 (12.5)	215 (11.5)	379 (11.0)		4700 (12.6)	594 (11.2)	
Education (n = 27,887)						<0.001			<0.001
High school graduate or higher	2649 (89.3)	6000 (85.1)	9319 (82.7)	825 (74.6)	1606 (75.2)		17,968 (84.6)	2431 (75.0)	
Less than high school	547 (10.7)	1901 (14.9)	3631 (17.3)	518 (25.4)	891 (24.8)		6079 (15.4)	1409 (25.0)	
Marital status (n = 27,642)						<0.001			<0.001
Married or living with a partner	2202 (72.3)	5478 (73.8)	8622 (71.1)	844 (66.7)	1517 (67.0)		16,302 (72.2)	2361 (66.9)	
Not married nor living with a partner	953 (27.7)	2354 (26.2)	4213 (28.9)	494 (33.3)	965 (33.0)		7520 (27.8)	1459 (33.1)	
Family income-to-poverty ratio (n = 25,576)						<0.001			<0.001
≥300%	1542 (64.6)	3217 (57.7)	5058 (56.6)	334 (42.9)	687 (43.0)		9817 (58.2)	1021 (43.0)	
<300%	1429 (35.4)	4032 (42.3)	6808 (43.4)	868 (57.1)	1601 (57.0)		12,269 (41.8)	2469 (57.0)	
Food security (n = 27,310)						<0.001			<0.001
Full security	2495 (86.7)	5600 (80.4)	9215 (80.7)	960 (81.3)	1609 (73.8)		17,310 (81.6)	2569 (75.9)	
Marginal, low, or very low security	634 (13.3)	2134 (19.6)	3458 (19.3)	363 (18.7)	842 (26.2)		6226 (18.4)	1205 (24.1)	
Type of health insurance (n = 27,822)						<0.001			<0.001
Private	2048 (73.1)	4713 (70.1)	7294 (67.8)	588 (54.2)	1025 (51.4)		14,055 (69.4)	1613 (52.2)	
Government or none	1136 (26.9)	3168 (29.9)	5629 (32.2)	755 (45.8)	1466 (48.6)		9933 (30.6)	2221 (47.8)	
Employment status (n = 27,896)						<0.001			<0.001
Employed, student, or retired	2571 (83.2)	6413 (84.6)	10,007 (82.2)	1095 (84.6)	1610 (69.8)		18,991 (83.2)	2705 (74.0)	
Unemployed	625 (16.8)	1487 (15.4)	2948 (17.8)	249 (15.4)	891 (30.2)		5060 (16.8)	1140 (26.0)	
Home ownership (n = 27,531)						<0.001			0.158
Own home	2103 (74.3)	5031 (71.7)	8809 (75.8)	965 (80.4)	1628 (73.3)		15,943 (74.2)	2593 (75.3)	
Rent home or other arrangement	1055 (25.7)	2765 (28.3)	3975 (24.2)	361 (19.6)	839 (26.7)		7795 (25.8)	1200 (24.7)	
Regular health-care access (n = 27,909)						<0.001			<0.001
At least one regular health-care facility	2560 (82.4)	6276 (82.5)	11,207 (88.6)	1278 (95.8)	2375 (95.6)		20,043 (85.5)	3653 (95.6)	
None or emergency room	637 (17.6)	1628 (17.5)	1755 (11.4)	66 (4.2)	127 (4.4)		4020 (14.5)	193 (4.4)	
Cumulative unfavorable SDOHs (n = 27,909)						<0.001			<0.001
<2	1600 (39.3)	4347 (43.9)	7215 (44.0)	886 (54.7)	1653 (55.4)		10,901 (56.8)	1307 (44.8)	
≥2	1597 (60.7)	3557 (56.1)	5747 (56.0)	458 (45.3)	849 (44.6)		13,162 (43.2)	2539 (55.2)	
Alcohol consumption (n = 23,572)						<0.001			<0.001
Non-drinker	636 (19.1)	1697 (21.3)	3172 (23.4)	348 (29.6)	644 (26.5)		5505 (22.0)	992 (27.3)	
Moderate to heavy drinker	2076 (80.9)	4868 (78.7)	7870 (76.6)	757 (70.4)	1504 (73.5)		14,814 (78.0)	2261 (72.7)	
Ever smoker (n = 27,888)						<0.001			<0.001
No	1815 (56.9)	4556 (56.6)	6817 (51.0)	505 (36.3)	863 (32.3)		13,188 (53.8)	1368 (33.4)	
Yes	1381 (43.1)	3341 (43.4)	6132 (49.0)	839 (63.7)	1639 (67.7)		10,854 (46.2)	2478 (66.6)	
Physical activity (n = 27,909)						<0.001			<0.001
Active	1617 (55.3)	3769 (50.8)	5271 (44.7)	411 (31.9)	834 (38.7)		10,657 (48.5)	1245 (36.8)	
Inactive	1580 (44.7)	4135 (49.2)	7691 (55.3)	933 (68.1)	1668 (61.3)		13,406 (51.5)	2601 (63.2)	
Anti-diabetes medication (n = 27,909)						NA			<0.001
No	3197 (100.0)	7904 (100.0)	11,349 (90.5)	683 (53.9)	1850 (78.2)		22,450 (95.3)	2533 (71.4)	
Yes	0 (0)	0 (0)	1613 (9.5)	661 (46.1)	652 (21.8)		1613 (4.7)	1313 (28.6)	
Anti-hypertensive medication (n = 27,909)						NA			<0.001
No	3197 (100.0)	7904 (100.0)	7414 (57.7)	262 (20.0)	877 (39.2)		18,515 (78.9)	1139 (33.8)	
Yes	0 (0)	0 (0)	5548 (42.3)	1082 (80.0)	1625 (60.8)		5548 (21.1)	2707 (66.2)	
Lipid-lowering medication (n = 14,898)						<0.001			<0.001
No	1025 (91.2)	2998 (91.8)	3803 (50.1)	242 (26.9)	410 (22.3)		7826 (66.8)	652 (23.6)	
Yes	111 (8.8)	261 (8.2)	3952 (49.9)	693 (73.1)	1403 (77.6)		4324 (33.2)	2096 (76.4)	

*Abbreviation*: SDOHs, social determinants of health.

a Data are presented as weighted mean (standard error) or weighted percentages.

b Differences in baseline characteristics across 5 cardiovascular-kidney-metabolic syndrome stages as well as between stages 3–4 and stages 0–2 were assessed using survey-weighted linear regression for continuous variables and survey-weighted Pearson’s chi-squared test for categorical variables.

c Other indicates other Hispanic or multi-racial.

### 3.2 CKM stages and premature mortality

During a median follow-up of 8.3 (interquartile range 4.4–12.8) years, there were 1762 premature deaths and 460 of them were due to CVD. Compared with CKM stage 0, the multivariable-adjusted HRs (95% CI) for all-cause premature mortality for stages 1–4 were 0.88 (95% CI 0.66–1.17), 1.31 (1.00–1.73), 1.96 (1.32–2.91), and 2.20 (1.62–2.99), respectively (Table 2). Similar results were observed when premature mortality was defined as deaths occurring before 65, 70, and 80 years of age (eTable 2). Compared with CKM stages 0–2 (non-advanced stages), the adjusted HR for all-cause premature mortality for stages 3–4 was 1.79 (1.53–2.10). For CVD premature mortality, the adjusted HRs (95% CI) for CKM stages 1–4 were 1.12 (0.46–2.72), 1.74 (0.71–4.28), 3.93 (1.53–10.12), and 6.48 (2.95–14.20), respectively (Table 2). The adjusted HR (95% CI) for CVD premature mortality for CKM stages 3–4 was 3.92 (2.86–5.39). For premature mortality from heart diseases, the adjusted HRs (95% CI) for CKM stages 1–4 were 0.99 (0.40–2.47), 1.76 (0.70–4.40), 4.43 (1.61–12.18), and 7.40 (3.40–16.13), respectively. For premature mortality from cerebrovascular diseases, the adjusted HRs (95% CI) for CKM stages 1–4 were 1.91 (0.34–10.84), 1.60 (0.38–6.74), 1.96 (0.24–16.32), and 2.46 (0.38–15.76), respectively (Table 2). After excluding the first 2 years of follow-up to minimize the reverse causation, the associations between CKM stages and premature death were not changed substantially (eTable 3). In the sensitivity analysis with competing risk models (Fine and Gray Cox regression models), similar results were observed (eTable 4). For the associations of CKM stages with all-cause or CVD premature mortality, there were no significant sex interaction and similar results were seen among females and males (Table 2).

**Table 2:** Adjusted hazard ratios for risk of premature mortality from all causes and cardiovascular disease according to cardiovascular–kidney–metabolic syndrome stages.

Outcome	Cardiovascular–kidney–metabolic syndrome stage^a^						
	0	1	2	3	4	Stages 0–2 (Non-advanced stages)	Stages 3–4 (Advanced stages)
Total population							
No. of participants	3197	7904	12,962	1344	2502	24,063	3846
Person years	34,135	77,118	115,187	6054	17,258	226,440	23,312
All deaths							
No. of deaths	115	251	849	133	414	1215	547
Rate of death, per 1000 person-years	3.4	3.3	7.4	22.0	24.0	5.4	23.5
Model 1^b^	1.00 (Reference)	0.89 (0.67–1.17)	1.55 (1.19–2.01)	2.66 (1.83–3.86)	3.62 (2.75–4.76)	1.00 (Reference)	2.54 (2.19–2.95)
Model 2^c^	1.00 (Reference)	0.88 (0.66–1.17)	1.31 (0.99–1.73)	1.94 (1.31–2.87)	2.19 (1.61–2.98)	1.00 (Reference)	1.79 (1.53–2.10)
Deaths from all cardiovascular diseases							
No. of deaths	21	44	203	32	160	268	192
Rate of death, per 1000 person-years	0.6	0.6	1.8	5.3	9.3	1.2	8.2
Model 1^b^	1.00 (Reference)	0.89 (0.43–1.84)	1.62 (0.81–3.24)	3.87 (1.65–9.08)	7.39 (3.93–13.88)	1.00 (Reference)	4.90 (3.63–6.62)
Model 2^c^	1.00 (Reference)	1.12 (0.46–2.72)	1.74 (0.71–4.28)	3.93 (1.53–10.12)	6.48 (2.95–14.20)	1.00 (Reference)	3.92 (2.86–5.39)
Deaths from heart diseases							
No. of deaths	18	36	162	27	139	216	166
Rate of death, per 1000 person-years	0.5	0.5	1.4	4.5	8.1	1.0	7.1
Model 1^b^	1.00 (Reference)	0.84 (0.39–1.80)	1.67 (0.80–3.49)	4.51 (1.76–11.56)	8.72 (4.52–16.83)	1.00 (Reference)	5.75 (4.16–7.97)
Model 2^c^	1.00 (Reference)	0.99 (0.40–2.47)	1.76 (0.70–4.40)	4.43 (1.61–12.18)	7.40 (3.40-16.13)	1.00 (Reference)	4.56 (3.28–6.35)
Deaths from cerebrovascular diseases							
No. of deaths	3	8	41	5	21	52	26
Rate of death, per 1000 person-years	0.1	0.1	0.4	0.8	1.2	0.2	1.1
Model 1^b^	1.00 (Reference)	1.15 (0.27–4.89)	1.34 (0.40–4.47)	1.64 (0.28–9.69)	2.46 (0.61–9.87)	1.00 (Reference)	1.78 (0.84–3.74)
Model 2^c^	1.00 (Reference)	1.91 (0.34–10.84)	1.60 (0.38–6.74)	1.96 (0.24–16.32)	2.46 (0.38–15.76)	1.00 (Reference)	1.46 (0.58–3.65)
Females							
No. of participants	1810	3986	6307	475	1036	12,103	1511
Person years	19,145	38,742	53,980	2174	7328	111,867	9502
All deaths							
No. of deaths	42	111	329	39	138	482	177
Rate of death, per 1000 person-years	2.2	2.9	6.1	17.9	18.8	4.3	18.6
Model 1^b^	1.00 (Reference)	1.17 (0.80–1.73)	1.84 (1.28–2.66)	4.99 (2.79–8.93)	5.45 (3.69–8.07)	1.00 (Reference)	3.36 (2.69–4.19)
Model 2^c^	1.00 (Reference)	1.01 (0.66–1.53)	1.33 (0.89–1.98)	2.79 (1.47–5.31)	2.54 (1.56–4.13)	1.00 (Reference)	2.08 (1.61–2.69)
Deaths from all cardiovascular diseases							
No. of deaths	8	13	71	13	45	92	58
Rate of death, per 1000 person-years							
Model 1^b^	1.00 (Reference)	0.67 (0.25–1.81)	1.61 (0.67–3.87)	12.57 (3.07–51.47)	10.07 (4.09–24.78)	1.00 (Reference)	7.98 (5.12–12.42)
Model 2^c^	1.00 (Reference)	0.63 (0.19–2.07)	1.37 (0.45–4.15)	10.06 (2.08–48.62)	7.02 (2.26–21.82)	1.00 (Reference)	6.36 (3.86–10.47)
Males							
No. of participants	1387	3918	6655	869	1466	11,960	2335
Person years	14,990	38,376	61,207	3880	9930	114,572	13,810
All deaths							
No. of deaths	73	140	520	94	276	733	370
Rate of death, per 1000 person-years	0.4	0.3	1.3	6.0	6.1	0.8	6.1
Model 1^b^	1.00 (Reference)	0.72 (0.51–1.01)	1.37 (1.00–1.86)	1.80 (1.16–2.80)	2.75 (1.97–3.85)	1.00 (Reference)	2.16 (1.80–2.60)
Model 2^c^	1.00 (Reference)	0.83 (0.58–1.18)	1.35 (0.98–1.86)	1.60 (1.03–2.50)	2.10 (1.48–2.98)	1.00 (Reference)	1.65 (1.34–2.03)
Deaths from all cardiovascular diseases							
No. of deaths	13	31	132	19	115	176	134
Rate of death, per 1000 person-years	0.7	0.8	2.4	8.7	15.7	1.6	14.1
Model 1^b^	1.00 (Reference)	0.98 (0.40–2.45)	1.59 (0.65–3.91)	1.73 (0.73–4.11)	6.13 (2.66–14.13)	1.00 (Reference)	3.81 (2.61–5.57)
Model 2^c^	1.00 (Reference)	1.34 (0.45–3.98)	1.86 (0.62–5.58)	1.81 (0.68–4.82)	5.91 (2.16–16.15)	1.00 (Reference)	3.10 (2.10–4.58)

a All estimates accounted for complex survey designs.

b Model 1 was adjusted for baseline age, sex, and race/ethnicity.

c Model 2 was further adjusted for education, marital status, family income-to-poverty ratio, food security, type of health insurance, employment status, home ownership, regular health-care access, alcohol consumption, smoking status, physical activity, and medical history of cancer at baseline.

### 3.3 CKM stages and all-cause premature mortality by SDOHs

**Fig. 1:**
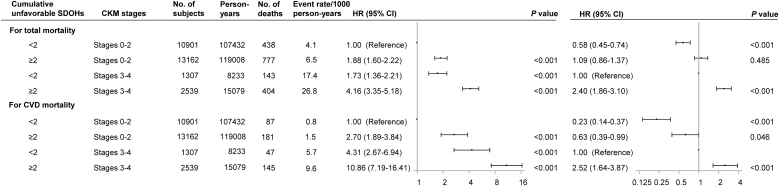
Adjusted hazard ratios for risk of premature mortality from all causes and cardiovascular disease according to cardiovascular–kidney–metabolic syndrome stages by cumulative unfavorable social determinants of health. *Abbreviations*: SDOHs, social determinants of health; CKM, cardiovascular–kidney–metabolic syndrome; HR, hazard ratio; CI, confidence interval. Points represent HRs and error bars represent 95% CIs. No adjustments of multiple comparisons were applied. All estimates accounted for complex survey designs. Models were adjusted for baseline age, sex, race/ethnicity, alcohol consumption, smoking status, physical activity, and medical history of cancer at baseline.

**Fig. 2:**
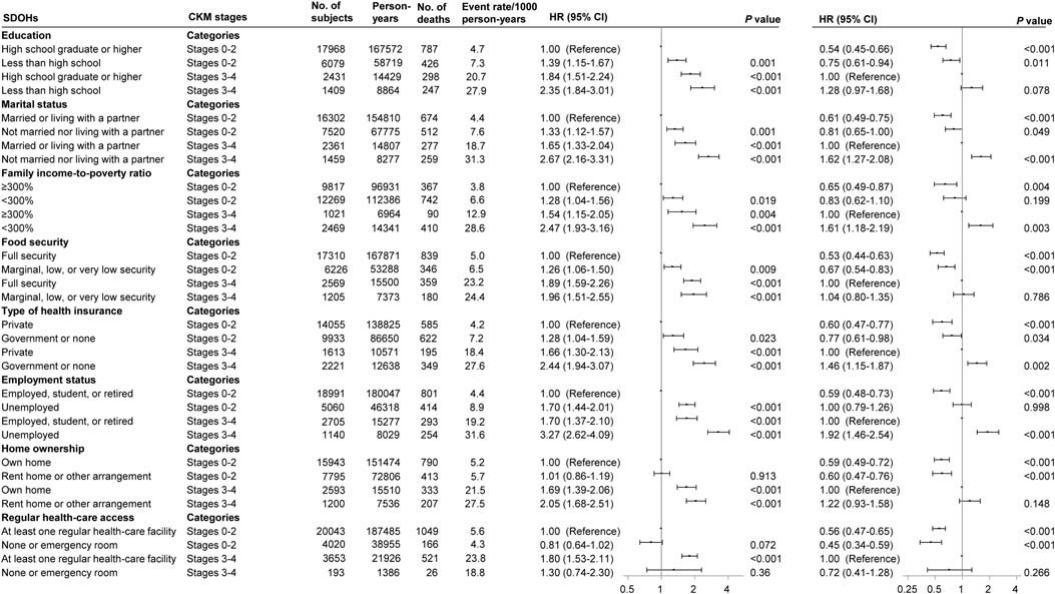
Adjusted hazard ratios for risk of all-cause premature mortality according to cardiovascular–kidney–metabolic syndrome stages by social determinants of health. *Abbreviations*: SDOHs, social determinants of health; CKM, cardiovascular–kidney–metabolic syndrome; HR, hazard ratio; CI, confidence interval. Points represent HRs and error bars represent 95% CIs. No adjustments of multiple comparisons were applied. All estimates accounted for complex survey designs. Models were adjusted for baseline age, sex, race/ethnicity, education, marital status, family income-to-poverty ratio, food security, type of health insurance, employment status, home ownership, regular health-care access, alcohol consumption, smoking status, physical activity, and medical history of cancer at baseline if applicable.

In multivariable-adjusted models, unfavorable SDOHs increased risks of all-cause premature mortality across CKM stages, with no interactions between SDOHs and CKM stages (*P* for interaction > 0.05 for all) (Fig. 1, Fig. 2 and eTable 5). Among participants at CKM stages 3–4, having 2 or more unfavorable SDOHs, not living with a partner, unemployment, low family income, and non private health insurance were associated with increased risks of all-cause premature mortality, while no significant associations of other SDOHs were observed (Fig. 1, Fig. 2). Having 2 or more unfavorable SDOHs was associated with higher risks of all-cause premature mortality than having fewer than 2 unfavorable SDOHs across all CKM stages (eTable 6). Among the 8 SDOHs, not married nor living with a partner and unemployment were associated with higher risks of all-cause premature mortality across CKM stages (eTable 6). Compared with CKM stage 0, stage 4 tended to be associated with increased all-cause premature mortality across different SDOH groups (eTable 7). Sex differences were observed and there was interaction of sex and employment status (*P* for interaction = 0.030) (eTables 8 and 9): among females at CKM stages 3–4 having 2 or more unfavorable SDOHs, a low education level, low family income, or unemployment was associated with higher all-cause premature mortality, while other SDOHs were not related to all-cause premature mortality; among males at CKM stages 3–4, having 2 or more unfavorable SDOHs, not living with a partner, low family income, non private health insurance, or unemployment was related to higher all-cause premature mortality, while there were no significant associations of other SDOHs. There were no significant interactions of age and specific SDOHs or cumulative unfavorable SDOHs (*P* for interaction > 0.05 for all) (eTables 10 and 11).

### 3.4 CKM stages and CVD premature mortality by SDOHs

**Fig. 3:**
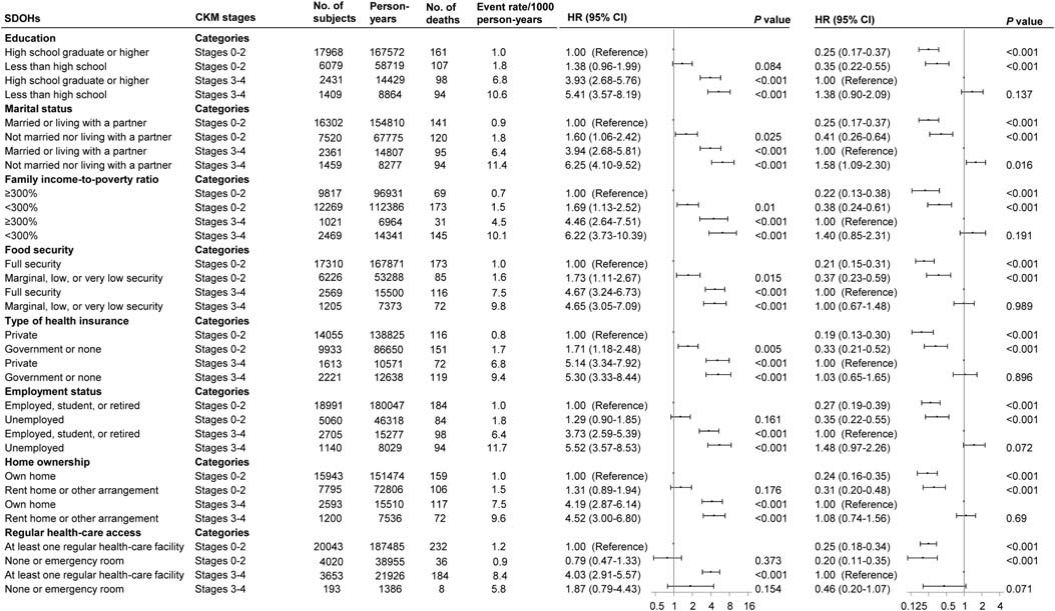
Adjusted hazard ratios for risk of premature mortality from cardiovascular disease according to cardiovascular–kidney–metabolic syndrome stages by social determinants of health. *Abbreviations*: SDOHs, social determinants of health; CKM, cardiovascular–kidney–metabolic syndrome; HR, hazard ratio; CI, confidence interval. Points represent HRs and error bars represent 95% CIs. No adjustments of multiple comparisons were applied. All estimates accounted for complex survey designs. Models were adjusted for baseline age, sex, race/ethnicity, education, marital status, family income-to-poverty ratio, food security, type of health insurance, employment status, home ownership, regular health-care access, alcohol consumption, smoking status, physical activity, and medical history of cancer at baseline if applicable.

In multivariable-adjusted models, unfavorable SDOHs increased risks of CVD premature mortality across CKM stages, with no interactions between SDOHs and CKM stages (*P* for interaction > 0.05 for all) (Fig. 1, Fig. 3 and eTable 12). Among participants at CKM stages 3–4, having 2 or more unfavorable SDOHs or not living with a partner was associated with increased risks of CVD premature mortality, while there were no significant associations of other SDOHs (Fig. 1, Fig. 3). In the sensitivity analysis with competing risk models (Fine and Gray Cox regression models), among participants at CKM stages 3–4, having 2 or more unfavorable SDOHs, not living with a partner, low family income, or unemployment was related to increased risks of CVD premature mortality (eTable 13). Compared with CKM stage 0, stage 4 tended to be associated with increased risks of CVD premature mortality across different SDOH groups (eTable 14). Having 2 or more unfavorable SDOHs was associated with higher risks of CVD premature mortality compared with having fewer than 2 unfavorable SDOHs among those at CKM stages 1–4 (eTable 15). Having none or emergency room was related to increased risks of CVD premature mortality than having at least 1 regular health-care facility among those at CKM stages 1–4. There were interactions of sex and family income (*P* for interaction = 0.002) or type of health insurance (*P* for interaction = 0.049) or home ownership (*P* for interaction = 0.049) or regular health-care access (*P* for interaction = 0.008) or cumulative unfavorable SDOHs (*P* for interaction = 0.016), but not education level or marital status or food security or employment status (eTables 16 and 17). Among females and males at CKM stages 3–4, having 2 or more unfavorable SDOHs increased the risk of CVD premature mortality, while individual SDOHs were not significantly associated with increased risks. There were interactions of age and type of health insurance (*P* for interaction = 0.026) or employment status (*P* for interaction = 0.034), but not other SDOHs or cumulative unfavorable SDOHs (eTables 18 and 19). Among middle-aged and older people at CKM stages 3–4, having government or none health insurance or unemployment increased the risk of CVD premature mortality.

## 4 Discussion

In this large prospective cohort study of US adults, we found that individuals at CKM stages 3–4 (advanced stages) had considerably higher risks of all-cause and CVD premature mortality than those without CKM risk (stage 0). Unfavorable SDOHs, especially not living with a partner, low family income, non-private health insurance, and unemployment, increased the risk of all-cause premature mortality among adults at CKM stages 3–4. Unfavorable SDOHs also increased premature mortality among those at stages 0–2 (non-advanced stages). In addition, evident sex differences were observed: low education level increased the risk among females only, while not living with a partner and not having private health insurance increased the risk among males only.

Globally, the burden of disease is projected to shift to non-communicable diseases between 2022 and 2050 [23]. Major non-communicable diseases, particularly components of CKM syndrome such as diabetes, CVD, and CKD are the leading cause of global deaths [23]. The combination of diabetes and CKD was associated with a higher 10-year mortality rate (31.1%) compared with diabetes or CKD alone (7.7% and 11.5%, respectively) in a nationwide sample [24]. A recent study has shown that CKM syndrome (stages 1–4) was associated with higher risks of all-cause mortality in Chinese people [8]. In addition to previous findings, in the present study, we provided new information that CKM stages 3–4, but not stages 1–2, were associated with higher all-cause and CVD premature mortality compared with stage 0. Our findings suggest that CKM stages 1 and 2 may represent a critical window for reducing the healthcare burden of CKM syndrome. Preventive action targeting individuals in these early stages could help prevent progression to more advanced stages (i.e., stages 3–4).

Many previous studies have demonstrated that unfavorable SDOHs were associated with CKM syndrome and its components such as diabetes, CVD, and CKD [12,13,[25], [26], [27], [28], [29]]. SDOHs were found to influence CVD and CKD mortality and there were socioeconomic disparities in mortality and life expectancy persist worldwide [30]. In England, approximately 35.6% of premature deaths were attributed to socioeconomic inequality [31]. Several previous studies have acknowledged associations between marital status and CVD risks [32]. Moreover, a meta-analysis of prospective cohort studies also suggested that compared with married individuals, being unmarried was associated with higher all-cause, cancer, and CVD mortality for both sexes [33]. Employment status and occupational categories are important markers of economic stability and some previous studies have revealed that these markers of economic stability, were independent determinants of CVD risk [9]. Findings from a prospective cohort study indicated that compared with employed individuals, the unemployed had a higher risk of all-cause mortality [34]. Similarly, increased risks of stroke and related mortality were observed in individuals who experienced job loss or reemployment in a prospective cohort study [35]. Our findings adds to the existing evidence by showing that specific unfavorable SDOHs e.g., not living with a partner, low family income, non-private health insurance, and unemployment increased the risk of all-cause premature mortality among adults at CKM stages 3–4. Our results strengthens the previous evidence and align with the US Healthy People 2030 initiative, which aims to achieve health equity with a key focus on improvements in SDOHs [36,37]. In addition, our findings underscore some certain SDOH components should be paid attention to for CKM and CVD prevention and treatment.

Multiple physiologic and behavioral mechanisms may explain the relationship between SDOHs and premature mortality from all causes and CVD. Low family income and unemployment have found to be linked with psychological stress and mental health disorders e.g., depression and anxiety [38,39]. Psychological stress and mental health disorders may have an impact on stress-induced hypothalamic activation and sympathetic nervous system, which may lead to vasoconstriction and increased peripheral vascular resistance [40]. These adverse physiological states are related to CVD risk factors e.g., high blood pressure, high heart rate, and low heart rate variability [40]. Socioeconomic disadvantages especially low education level are associated with unhealthy lifestyle e.g., unhealthy diet, physical inactivity, irregular sleep, and smoking, which may induce metabolic disorders and in turn related to CVD and mortality [9].

The role of sex in CKM health has been investigated by previous studies. A recent Chinese cohort study found that low education level was related to adverse CKM health for both sexes but was especially detrimental to females [13]. In the current study, we further explored the role of sex in the relationship between CKM stages and premature mortality. We observed sex differences and showed that for females at CKM stages 3–4, low education level, low family income, or unemployment increased the risk of all-cause premature mortality, while among males at CKM stages 3–4, not living with a partner, low family income, non-private health insurance, or unemployment increased the risk.

Our findings suggest that adults at CKM stages 3–4 had increased risks of premature mortality and therefore it would be necessary for those at stages 0–2 to prevent from progression to advanced stages via lifestyle modification or other therapies. Moreover, our study implies that SDOHs had an important role to play, and it may be important to integrate screening and data collection of SDOHs into electronic health records and clinical workflows. Interventions targeting SDOHs, particularly sex- and age-specific interventions, to reduce premature mortality among individuals at CKM stages 3–4 may be taken into consideration.

This study has several limitations. First, as this was an observational study, residual and unmeasured confounding factors such as genes were not able to be ruled out, preventing us from concluding a causal association between CKM stages and premature mortality. Second, premature death occurred near CKM stage ascertainment may distorted the CKM categories and thus produce reverse causation. However, after excluding the first 2 years of follow-up, the associations between CKM stages and premature death were not changed substantially. Third, some data recommended by the AHA for defining CKM stages 3–4 such as cardiac biomarkers, cardiac computed tomography, atrial fibrillation, coronary angiography, and peripheral artery disease, were not available in the NHANES database, which may have led to an underestimation of stages 3 and 4. Fourth, SDOHs were self-reported, potentially introducing misclassification bias. Additionally, while SDOHs include the economic, social, environmental, and psychosocial factors, variables related to social cohesion, structural racism, or neighborhood and community environments were not available in the NHANES database, limiting our ability to assess the role of these variables in the current study. Fifth, we did not investigate the sex-specific differences in the relationship between each CKM stage and premature mortality, because we did not have sufficient sample size and the number of events by sex at each CKM stage, especially stage 3. Furthermore, adjustments for multiple comparisons were not applied in this study, which may cause type I error, and the findings therefore should be interpreted as exploratory. Finally, as the PREVENT equations were not applicable for adults aged <30 or >79 years and those without extreme values of CVD risk factors, we had to limit participants’ age to 30–79 years and exclude those with extreme values, which may affect the generalizability of our findings.

## 5 Conclusion

In conclusion, adults at CKM stages 3–4 (advanced stages), but not stages 1–2 had higher risks of all-cause and CVD premature mortality compared with stage 0. The risks of premature mortality might be increased by unfavorable SDOHs across CKM stages. Among those with unfavorable SDOHs, not living with a partner, low family income, non-private health insurance, and unemployment increased the risk of all-cause premature mortality among adults at CKM stages 3–4. These findings suggested that CKM stages 1–2 might represent a critical window of opportunity to reduce the CKM burden. Moreover, interventions targeting SDOHs, particularly sex- and age-specific interventions, may be needed for prevention of premature mortality associated with CKM stages 3–4.

## CRediT authorship contribution statement

RZ, JG, RW, and JH contributed to the study conception and design. RZ and JG drafted the data analysis plan. JG analyzed the data. RZ and JG wrote the manuscript. RW, JH, LW, HC, YW, PA, KL, FR, WX, J.AM, and AR critically revised the manuscript for important intellectual content. All authors commented on the drafts and approved the final draft. JG and RZ are the guarantors. The corresponding author (JG) attests that all listed authors meet authorship criteria and that no others meeting the criteria have been omitted. JG and RZ had full access to all the data in the study and take responsibility for the integrity of the data and the accuracy of the data analysis.

## Ethics approval and consent to participate

The Institutional Review Board for the National Center for Health Statistics (now referred to as the Ethics Review Board) approved the NHANES protocol and allowed the data files to be posted on their website for public use. All participants provided written informed consent before data collection.

The content is solely the responsibility of the authors.

## Declaration of Generative AI and AI-assisted technologies in the writing process

The authors did not use any AI at all in the writing process.

## Funding/support

This research was supported by Stiftelser förvaltade av Lindhés Advokatbyrå AB (LA2023-0065), the 2115 Talent Development Program of 10.13039/501100002365China Agricultural University, and the China Postdoctoral Science Foundation (Grant numbers: 2023T00376 and 2023M743787).

The funders/sponsors had no role in design and conduct of the study; collection, management, analysis, or interpretation of the data; preparation, review, or approval of the manuscript; or decision to submit the manuscript for publication.

## Declaration of competing interest

AR has received honorariums from the International Sweeteners Association, Nestlé and Unilever and research funds from Novo Nordisk A/S. Other authors have no conflict of interest to disclose.

## Data availability

All relevant data in this study are publicly available in National Health and Nutrition Examination Survey Homepage (https://www.cdc.gov/nchs/nhanes/index.htm). All-cause mortality was ascertained via linkage with the National Death Index (https://www.cdc.gov/nchs/ndi/index.htm). The corresponding author Dr Jie Guo (email address: jie.guo@ki.se) should be contacted for any requests (e.g., data used for all analyses; analytic code; any other materials used in the current study).
