# Supplementary material for: Associations of cardiovascular–kidney–metabolic syndrome stages with premature mortality and the role of social determinants of health
Source: J Nutr Health Aging. 2025 Feb 13;29(4):100504. doi: 10.1016/j.jnha.2025.100504 (PMC12180020; doi:10.1016/j.jnha.2025.100504)
Supplement: Supplementary file 1 [file mmc1.docx]

**Supplemental Material**

**eMethods**

**eFigure 1.** Flow chart of the study population

**eTable 1.** Definitions of cardiovascular-kidney-metabolic syndrome stages

**eTable 2.** Adjusted hazard ratios for risk of premature mortality from all causes and cardiovascular disease according to cardiovascular-kidney-metabolic syndrome stages

**eTable 3.** Adjusted hazard ratios for risk of premature mortality from all causes and cardiovascular disease according to cardiovascular-kidney-metabolic syndrome stages after excluding the first 2 years of follow-up

**eTable 4.** Adjusted hazard ratios for risk of premature mortality from cardiovascular disease according to cardiovascular-kidney-metabolic syndrome stages: Fine and Gray Cox regression models

**eTable 5.** Adjusted hazard ratios for risk of all-cause premature mortality according to cardiovascular-kidney-metabolic syndrome stages, with stage 0 in each subgroup of age, sex, and social determinants of health as reference

**eTable 6.** Adjusted hazard ratios for risk of all-cause premature mortality according to cardiovascular-kidney-metabolic syndrome stages, with a subgroup of social determinants of health as reference

**eTable 7.** Adjusted hazard ratios for risk of all-cause premature mortality according to cardiovascular-kidney-metabolic syndrome stages by social determinants of health

**eTable 8.** Adjusted hazard ratios for risk of all-cause premature mortality according to cardiovascular-kidney-metabolic syndrome stages by social determinants of health among females and males, with stages 0-2 as reference

**eTable 9.** Adjusted hazard ratios for risk of all-cause premature mortality according to cardiovascular-kidney-metabolic syndrome stages by social determinants of health among females and males, with stages 3-4 as reference

**eTable 10.** Adjusted hazard ratios for risk of premature mortality from all causes according to cardiovascular-kidney-metabolic syndrome stages by social determinants of health across age groups, with stages 0-2 as reference

**eTable 11.** Adjusted hazard ratios for risk of premature mortality from all causes according to cardiovascular-kidney-metabolic syndrome stages by social determinants of health across age groups, with stages 3-4 as reference

**eTable 12.** Adjusted hazard ratios for risk of premature mortality from cardiovascular disease according to cardiovascular-kidney-metabolic syndrome stages, with stage 0 in each subgroup of age, sex, and social determinants of health as reference

**eTable 13.** Adjusted hazard ratios for risk of premature mortality from cardiovascular disease according to cardiovascular-kidney-metabolic syndrome stages by social determinants of health: Fine and Gray Cox regression models

**eTable 14.** Adjusted hazard ratios for risk of premature mortality from cardiovascular disease according to cardiovascular-kidney-metabolic syndrome stages by social determinants of health

**eTable 15.** Adjusted hazard ratios for risk of premature mortality from cardiovascular disease according to cardiovascular-kidney-metabolic syndrome, with a subgroup of social determinants of health as reference

**eTable 16.** Adjusted hazard ratios for risk of premature mortality from cardiovascular disease according to cardiovascular-kidney-metabolic syndrome stages by social determinants of health among females and males, with stages 0-2 as reference

**eTable 17.** Adjusted hazard ratios for risk of premature mortality from cardiovascular disease according to cardiovascular-kidney-metabolic syndrome stages by social determinants of health among females and males, with stages 3-4 as reference

**eTable 18.** Adjusted hazard ratios for risk of premature mortality from cardiovascular disease according to cardiovascular-kidney-metabolic syndrome stages by social determinants of health across age groups, with stages 0-2 as reference

**eTable 19.** Adjusted hazard ratios for risk of premature mortality from cardiovascular disease according to cardiovascular-kidney-metabolic syndrome stages by social determinants of health across age groups, with stages 3-4 as reference

**eReferences**

**eMethods**

**Assessment of cardiovascular-kidney-metabolic syndrome stages**

According to the American Heart Association (AHA) criteria, we classified cardiovascular-kidney-metabolic (CKM) syndrome into 5 stages (i.e., stages 0-4). CKM stage 0 identified participants with normal body mass index (BMI<25 kg/m^2^) and normal waist circumference (<88/102 cm in women/men) who did not meet criteria for the other stages; stage 1 identified participants with an elevated BMI (≥25 kg/m^2^), an elevated waist circumference (<88/102 cm in women/men), or prediabetes (fasting blood glucose 100-125 mg/dL, glycosylated hemoglobin A_1c_ [HbA_1c_] 5.7%-6.4%); stage 2 identified participants with metabolic risk factors, including elevated fasting serum triglycerides (≥135 mg/dL), hypertension (systolic blood pressure≥140 mmHg, diastolic blood pressure≥90 mmHg), diabetes (fasting blood glucose≥126 mg/dL or HbA_1c_≥6.5% or use of anti-diabetes treatment), or metabolic syndrome (≥3 of the following: elevated waist circumference, low high density lipoprotein cholesterol [HDL] level [<40 mg/dL or <50 mg/dL for men or women, respectively], fasting serum triglycerides≥150 mg/dL, elevated blood pressure [systolic blood pressure≥130 mmHg, diastolic blood pressure≥80 mmHg, and/or use of blood pressure-lowering medications]) or moderate-to-high-risk chronic kidney disease (CKD) according to Kidney Disease Improving Global Outcomes (KDIGO) criteria [1]; stage 3 identified participants with very-high-risk KDIGO CKD or a high-predicted 10-year CKD risk; stage 4 identified participants with self-reported cardiovascular disease (CVD) (i.e. coronary heart disease, angina, heart attack, heart failure, and stroke). We estimated 10-year CVD risk using the AHA Predicting Risk of CVD EVENTs (PREVENT) equation (base model) [2] and high risk of 10-year CVD risk was defined as PREVENT≥20%. The PREVENT equations were developed by the AHA CKM Scientific Advisory Group and used to estimate 10-year CVD for individuals 30-79 years of age. The PREVENT equations were derived and validated in a large, diverse sample of over 6 million individuals. The risk equations were based on base models and add-on models. The base models incorporate sex (female or male), age, total cholesterol, HDL cholesterol, systolic blood pressure, BMI, estimated glomerular filtration rate (eGFR), diabetes (yes or no), current smoking (yes or no), anti-hypertensive medication (yes or no), and lipid-lowering medication (yes or no). Add-on models further incorporate HbA_1c_, urine albumin-to-creatinine ratio, and social deprivation index. This study used the base model for 10-year CVD risk estimation. CKD stages were identified based on glomerular filtration rate and urinary albumin-to-creatinine ratio. eGFR was calculated with the race-free CKD-EPI 2021 creatinine equation [3].

**Assessment of covariates**

Age was categorized into <60 and ≥ 60 years. Race/ethnicity included Mexican American, non-Hispanic Black, non-Hispanic White, and other (other Hispanic and other race - including multi-racial) and was dichotomized into White and non-White. Cancer at baseline was identified based on self-reported cancer history except non-melanoma skin cancer. Smoking status was categorized as ever (i.e., current or former smoker) and never smoker. Alcohol consumption was dichotomized as drinker if participants self-reported that they had at least 12 alcohol drinks per year or non-drinker if they had less than 12 alcohol drinks per year. Regarding physical activity, participants were asked to report the intensity level (i.e., moderate- or vigorous-intensity), frequency, and duration of physical activity during the past 30 days. In addition, they were asked to report their engagement, frequency, and duration in daily activities (i.e., walking, bicycling, and tasks around home/yard) during the past 30 days. The NHANES guidelines suggested a metabolic equivalent score of 4.0 for one minute for walking or bicycling and a metabolic equivalent score of 4.5 for tasks around home/yard. These activities were thus defined as being of moderate-intensity (3.0 to 5.9 metabolic equivalents). Together, total minutes per week of moderate- and vigorous-intensity physical activity were calculated. Physical activity was categorized as active (≥150/75 min/week for moderate/vigorous-intensity activity) or inactive. We included 8 social determinants of health (SDOHs) available and they were dichotomized (favorable versus unfavorable) based on conventional cutoff points: employment status (employed, student, or retired versus unemployed), family income-to-poverty ratio (≥300% versus <300%), food security (full security versus marginal, low, or very low security), education level (high school graduate or higher versus less than high school), health-care access (at least 1 regular health-care facility versus none or emergency room), health insurance status (private versus government or no insurance), home ownership (own home versus rent home or other arrangement), and marital status (married or living with a partner versus not married or living with a partner) [4]. The cumulative unfavorable SDOHs was calculated by summing the 8 dichotomized SDOHs, assigning a value of 1 for each unfavorable level and 0 for each favorable level and were further dichotomized as ≥2 versus <2 favorable SDOHs based on the median level [4]. Because misreporting of energy and nutrient intake, especially under-reporting, remains prevalent in US adults involved in NHANES, we did not include dietary factors in this study [5, 6].

**eFigure 1. Flow chart of the study population**

Abbreviations: BMI, body mass index; eGFR, estimated glomerular filtration rate; HbA_1c_, glycosylated hemoglobin A_1c_; HDL, high-density lipoprotein cholesterol; SBP, systolic blood pressure; TC, total cholesterol.

101,316 participants from NHANES 1999-2018

38,018 participants aged 30-74 years

Excluding 2073 participants who were pregnant or lactating

27,909 participants aged 30-74 years at baseline

Excluding 61,225 participants aged<30 or ≥75 years

Excluding 3373 participants with missing SBP, SBP<90, or SBP>200 mmHg

Excluding 2991 participants with missing TC, TC<130, or TC>320 mg/dL

Excluding 395 participants with missing HDL, HDL<20, or HDL>100 mg/dL

Excluding 190 participants with missing eGFR, eGFR<15, or >140 mL/min/1.73m^2^

Excluding 2894 participants with missing BMI, BMI<18.5, or >39.9 kg/m^2^

Excluding 207 participants with missing HbA_1c_, HbA_1c_<4.5%, or HbA1c>15%

Excluding 59 participants without eligible death information

**eTable 1. Definitions of cardiovascular-kidney-metabolic syndrome stages**

| CKM stages | Definition according to the AHA statement | NHANES adapted definition according to Aggarwal et al |
| --- | --- | --- |
| **Stage 0:**  No CKM risk factors | Normal BMI, and  Normal waist circumference (<88/102 cm in women/men), and  Normoglycemia (fasting blood glucose<100 mg/dL or  glycated hemoglobin A_1c_<5.7%), and  Normotension (systolic blood pressure<130 mmHg or diastolic blood pressure<80 mmHg), and  Normal lipid profile, and  No evidence of CKD or subclinical or clinical CVD | Normal BMI, and  Normal waist circumference (<88/102 cm in women/men), and  Participants who did not meet criteria for the other stages |
| **Stage 1:**  Excess or dysfunctional adiposity | Overweight/obesity (BMI≥25 kg/m^2^, or ≥23 kg/m^2^ if Asian ancestry), or  Abdominal obesity (waist circumference≥88/102 cm in women/men, or if Asian ancestry≥80/90 cm in women/men), or  Dysfunctional adipose tissue, or  Without the presence of other metabolic risk factors (fasting blood glucose 100-125 mg/dL or  glycated hemoglobin A_1c_ 5.7%-6.4%) or  CKD | Elevated BMI (BMI≥25 kg/m^2^, or ≥23 kg/m^2^ if Asian ancestry), or  Elevated waist circumference (waist circumference≥88/102 cm in women/men, or if Asian ancestry≥80/90 cm in women/men), or  Prediabetes (defined as a glycated hemoglobin A_1c_ of 5.7% to <6.5% or a fasting blood glucose of 100 mg/dL to <126 mg/dL) |
| **Stage 2:**  Metabolic risk factors and CKD | Metabolic risk factors (fasting serum triglycerides ≥135 mg/dL, hypertension: systolic blood pressure ≥130 mmHg or diastolic blood pressure ≥80 mmHg and/or use of antihypertensive medications, diabetes: fasting blood glucose≥126 mg/dL or glycated hemoglobin A_1c_≥6.5% or use of anti-diabetes treatment, MetS), or CKD  MetS is defined by the presence of 3 or more of the following: (1) waist circumference≥88 cm for women and ≥102 cm for men (≥80 cm for women and ≥90 cm for men if Asian ancestry); (2) HDL cholesterol<40 mg/dL for men and <50 mg/dL for women; (3) triglycerides≥150 mg/dL; (4) elevated blood pressure (systolic blood pressure≥130 mmHg or diastolic blood pressure≥80 mmHg and/or use of antihypertensive medications); and (5) fasting blood glucose≥100 mg/dL | Metabolic risk factors, or moderate-to-high-risk CKD per Kidney Disease Improving Global Outcomes (KDIGO) criteria, as recommended by the AHA  Qualifying metabolic risk factors included elevated fasting serum triglycerides (≥135 mg/dL), hypertension, diabetes (fasting blood glucose≥126 mg/dL or glycated hemoglobin A_1c_≥6.5% or use of anti-diabetes treatment), or metabolic syndrome (≥3 of the following: elevated waist circumference, low high density lipoprotein cholesterol (HDL) level [<40 mg/dL or <50 mg/dL for men or women, respectively], fasting serum triglycerides≥150 mg/dL, elevated blood pressure [systolic blood pressure≥130, diastolic blood pressure≥80 mmHg, and/or use of blood pressure-lowering medications])  CKD stages were identified based on GFR and urinary albumin-to-creatinine ratio |
| **Stage 3:**  Subclinical CVD in CKM | Subclinical ASCVD or subclinical heart failure among individuals with excess/dysfunctional adiposity, other metabolic risk factors, or CKD   1. Subclinical ASCVD to be principally diagnosed by coronary artery calcification (subclinical atherosclerosis by coronary catheterization/CT angiography also meets criteria) 2. Subclinical heart failure diagnosed by elevated cardiac biomarkers (NT-proBNP≥125 pg/mL, hs-troponin T≥14 ng/L for women and ≥22 ng/L for men, hs-troponin I≥10 ng/L for women and ≥12 ng/L for men) or by echocardiographic parameters, with a combination of the 2 indicating highest HF risk   Risk equivalents of subclinical CVD   1. Very high-risk CKD (stage G4 or G5 CKD or very high risk per Kidney Disease Improving Global Outcomes (KDIGO) classification) 2. High predicted 10-year CVD risk | The presence of very-high-risk KDIGO CKD stages or a high-predicted 10-year CVD risk. 10-year cardiovascular risk was estimated with the AHA Predicting Risk of CVD EVENTs (PREVENT) equations. High risk was defined as ≥20% 10-year CVD risk (based on recommended thresholds[https://professional.heart.org/en/guidelines-and-statements/prevent-calculator]). The PREVENT equations were developed and validated for adults 30-79 years of age. As such, risk was not estimated for adults <30 years. However, to minimize underestimation of CKD Stage 3, adults≥80 years were not excluded from 10-year CVD risk. Instead, adults≥80 years were assigned an age of 79 years when determining 10-year CVD risk to allow for conservative estimates. Further, PREVENT was developed for variables with the following ranges: total cholesterol 130-320 mg/dL, HDL 20-100 mg/dL, systolic blood pressure 90-200 mmHg, and GFR 14-140 mL/min/1.73m^2^. To approximate PREVENT risk strata, values for these variables above or below these bounds were set to the upper or lower bounds of allowable values respectively (for example, total cholesterol of 330 mg/dL was set as 320 mg/dL). Cardiac biomarkers and cardiovascular imaging were not available to identify subclinical CVD |
| **Stage 4:**  Clinical CVD in CKM | Clinical CVD (coronary heart disease, heart failure, stroke, peripheral artery disease, atrial fibrillation) among individuals with excess/dysfunctional adiposity, other CKM risk factors, or CKD  Stage 4a: no kidney failure  Stage 4b: kidney failure present | Self-reported established cardiovascular disease (coronary heart disease, angina, heart attack, heart failure, and stroke). Atrial fibrillation and peripheral artery disease were not included, as these data were not available |

Abbreviations: ASCVD, atherosclerotic cardiovascular disease; BMI, body mass index; CKD, chronic kidney disease; CKM, cardiovascular-kidney-metabolic syndrome; CVD, cardiovascular disease; GFR, glomerular filtration rate; HDL, high-density lipoprotein cholesterol; MetS, metabolic syndrome

**eTable 2. Adjusted hazard ratios for risk of premature mortality from all causes and cardiovascular disease according to cardiovascular-kidney-metabolic syndrome stages**

| Outcome | Cardiovascular-kidney-metabolic syndrome stage^a^ | | | | | | |
| --- | --- | --- | --- | --- | --- | --- | --- |
|  | 0 | 1 | 2 | 3 | 4 | Stages 0-2 (Non-advanced stages) | Stages 3-4 (Advanced stages) |
| **Premature death using 65 years as cut-off** |  |  |  |  |  |  |  |
| No. of participants | 3009 | 7402 | 10,529 | 270 | 1392 | 20,940 | 1662 |
| Person years | 31,069 | 68,744 | 83,234 | 1090 | 8615 | 183,047 | 9705 |
| All deaths |  |  |  |  |  |  |  |
| No. of deaths | 72 | 165 | 394 | 18 | 127 | 631 | 145 |
| Rate of death, per 1000 person-years | 2.3 | 2.4 | 4.7 | 16.5 | 14.7 | 3.4 | 14.9 |
| Model 1^b^ | 1.00 (Reference) | 1.03 (0.74-1.44) | 1.81 (1.31-2.49) | 6.64 (3.22-13.69) | 4.45 (3.09-6.41) | 1.00 (Reference) | 3.14 (2.48-3.98) |
| Model 2^c^ | 1.00 (Reference) | 1.01 (0.71-1.44) | 1.50 (1.07-2.11) | 4.64 (2.22-9.70) | 2.30 (1.55-3.40) | 1.00 (Reference) | 1.87 (1.46-2.40) |
| Deaths from cardiovascular disease |  |  |  |  |  |  |  |
| No. of deaths | 8 | 27 | 95 | 6 | 53 | 130 | 59 |
| Rate of death, per 1000 person-years | 0.3 | 0.4 | 1.1 | 5.5 | 6.2 | 0.7 | 6.1 |
| Model 1^b^ | 1.00 (Reference) | 1.37 (0.53-3.53) | 3.08 (1.17-8.05) | 25.63 (5.78-113.6) | 14.71 (5.72-37.80) | 1.00 (Reference) | 6.84 (4.33-10.80) |
| Model 2^c^ | 1.00 (Reference) | 1.70 (0.58-4.95) | 3.32 (1.15-9.57) | 28.82 (6.90-120.4) | 12.20 (4.45-33.46) | 1.00 (Reference) | 5.19 (3.38-7.97) |
| **Premature death using 70 years as cut-off** |  |  |  |  |  |  |  |
| No. of participants | 3118 | 7714 | 12,000 | 660 | 1928 | 22,832 | 2588 |
| Person years | 32,898 | 73,786 | 100,798 | 2723 | 12,638 | 207,482 | 15,361 |
| All deaths |  |  |  |  |  |  |  |
| No. of deaths | 95 | 214 | 602 | 52 | 244 | 911 | 296 |
| Rate of death, per 1000 person-years | 2.9 | 2.9 | 6.0 | 19.1 | 19.3 | 4.4 | 19.3 |
| Model 1^b^ | 1.00 (Reference) | 0.96 (0.71-1.28) | 1.61 (1.22-2.14) | 3.84 (2.33-6.32) | 3.87 (2.93-5.12) | 1.00 (Reference) | 2.80 (2.36-3.33) |
| Model 2^c^ | 1.00 (Reference) | 0.95 (0.70-1.30) | 1.35 (1.01-1.82) | 2.95 (1.73-5.03) | 2.25 (1.64-3.09) | 1.00 (Reference) | 1.90 (1.59-2.26) |
| Deaths from cardiovascular disease |  |  |  |  |  |  |  |
| No. of deaths | 16 | 35 | 138 | 13 | 101 | 189 | 114 |
| Rate of death, per 1000 person-years | 0.5 | 0.5 | 1.4 | 4.8 | 8.0 | 0.9 | 7.4 |
| Model 1^b^ | 1.00 (Reference) | 0.90 (0.42-1.93) | 1.64 (0.78-3.44) | 6.56 (2.13-20.22) | 8.70 (4.49-16.86) | 1.00 (Reference) | 6.20 (4.39-8.74) |
| Model 2^c^ | 1.00 (Reference) | 1.18 (0.47-2.94) | 1.71 (0.68-4.31) | 7.26 (2.25-23.43) | 7.44 (3.34-16.60) | 1.00 (Reference) | 4.93 (3.53-6.89) |
| **Premature death using 80 years as cut-off** |  |  |  |  |  |  |  |
| No. of participants | 3216 | 7969 | 13,202 | 2264 | 3008 | 24,387 | 5272 |
| Person years | 34,915 | 79,123 | 124,718 | 11,623 | 21,890 | 238,756 | 33,514 |
| All deaths |  |  |  |  |  |  |  |
| No. of deaths | 133 | 302 | 1076 | 335 | 660 | 1511 | 995 |
| Rate of death, per 1000 person-years | 3.8 | 3.8 | 8.6 | 28.8 | 30.2 | 6.3 | 29.7 |
| Model 1^b^ | 1.00 (Reference) | 0.90 (0.69-1.16) | 1.45 (1.15-1.83) | 2.37 (1.73-3.24) | 3.49 (2.70-4.49) | 1.00 (Reference) | 2.43 (2.13-2.78) |
| Model 2^c^ | 1.00 (Reference) | 0.87 (0.66-1.15) | 1.24 (0.96-1.59) | 1.76 (1.26-2.46) | 2.30 (1.74-3.04) | 1.00 (Reference) | 1.87 (1.63-2.13) |
| Deaths from cardiovascular disease |  |  |  |  |  |  |  |
| No. of deaths | 26 | 48 | 264 | 87 | 248 | 338 | 335 |
| Rate of death, per 1000 person-years | 0.7 | 0.6 | 2.1 | 7.5 | 11.3 | 1.4 | 10.0 |
| Model 1^b^ | 1.00 (Reference) | 0.85 (0.44-1.63) | 1.63 (0.92-2.89) | 3.66 (1.94-6.90) | 6.97 (4.07-11.93) | 1.00 (Reference) | 4.41 (3.34-5.81) |
| Model 2^c^ | 1.00 (Reference) | 0.95 (0.45-2.00) | 1.64 (0.81-3.31) | 3.27 (1.62-6.57) | 5.93 (3.15-11.19) | 1.00 (Reference) | 3.65 (2.74-4.87) |

^a^ All estimates accounted for complex survey designs.

^b^ Model 1 was adjusted for baseline age, sex, and race/ethnicity.

^c^ Model 2 was further adjusted for education, marital status, family income-to-poverty ratio, food security, type of health insurance, employment status, home ownership, regular health-care access, alcohol consumption, smoking status, physical activity, and medical history of cancer at baseline.

**eTable 3. Adjusted hazard ratios for risk of premature mortality from all causes and cardiovascular disease according to cardiovascular-kidney-metabolic syndrome stages after excluding the first 2 years of follow-up** ^a^

|  | Full sample | |  | Excluding first 2 years of follow-up | |
| --- | --- | --- | --- | --- | --- |
|  | Stages 0-2 (Non-advanced stages) | Stages 3-4 (Advanced stages) |  | Stages 0-2 (Non-advanced stages) | Stages 3-4 (Advanced stages) |
| **All deaths** |  |  |  |  |  |
| Model 1^b^ | 1.00 (Reference) | 2.54 (2.19-2.95) |  | 1.00 (Reference) | 2.53 (2.16-2.97) |
| Model 2^c^ | 1.00 (Reference) | 1.79 (1.53-2.10) |  | 1.00 (Reference) | 1.80 (1.52-2.13) |
| **Deaths from cardiovascular disease** |  |  |  |  |  |
| Model 1^b^ | 1.00 (Reference) | 4.90 (3.63-6.62) |  | 1.00 (Reference) | 4.71 (3.40-6.51) |
| Model 2^c^ | 1.00 (Reference) | 3.92 (2.86-5.39) |  | 1.00 (Reference) | 3.70 (2.58-5.33) |

^a^ All estimates accounted for complex survey designs.

^b^ Model 1 was adjusted for baseline age, sex, and race/ethnicity.

^c^ Model 2 was further adjusted for education, marital status, family income-to-poverty ratio, food security, type of health insurance, employment status, home ownership, regular health-care access, alcohol consumption, smoking status, physical activity, and medical history of cancer at baseline.

**eTable 4. Adjusted hazard ratios for risk of premature mortality from cardiovascular disease according to cardiovascular-kidney-metabolic syndrome stages: Fine and Gray Cox regression models** ^a^

| Outcome | Cardiovascular-kidney-metabolic syndrome stage^a^ | | | | | | |
| --- | --- | --- | --- | --- | --- | --- | --- |
|  | 0 | 1 | 2 | 3 | 4 | Stages 0-2 (Non-advanced stages) | Stages 3-4 (Advanced stages) |
| No. of participants | 3197 | 7904 | 12,962 | 1344 | 2502 | 24,063 | 3846 |
| Person years | 34,135 | 77,118 | 115,187 | 6054 | 17,258 | 226,440 | 23,312 |
| **Deaths from all cardiovascular diseases** |  |  |  |  |  |  |  |
| No. of deaths | 21 | 44 | 203 | 32 | 160 | 268 | 192 |
| Rate of death, per 1000 person-years | 0.6 | 0.6 | 1.8 | 5.3 | 9.3 | 1.2 | 8.2 |
| Model 1^b^ | 1.00 (Reference) | 0.89 (0.67-1.17) | 1.55 (1.19-2.01) | 2.66 (1.83-3.86) | 3.62 (2.75-4.76) | 1.00 (Reference) | 2.54 (2.19-2.95) |
| Model 2^c^ | 1.00 (Reference) | 0.88 (0.66-1.17) | 1.31 (0.99-1.73) | 1.94 (1.31-2.87) | 2.19 (1.61-2.98) | 1.00 (Reference) | 1.79 (1.53-2.10) |

^a^ All estimates accounted for complex survey designs.

^b^ Model 1 was adjusted for baseline age, sex, and race/ethnicity.

^c^ Model 2 was further adjusted for education, marital status, family income-to-poverty ratio, food security, type of health insurance, employment status, home ownership, regular health-care access, alcohol consumption, smoking status, physical activity, and medical history of cancer at baseline.

**eTable 5. Adjusted hazard ratios for risk of all-cause premature mortality according to cardiovascular-kidney-metabolic syndrome stages, with stage 0 in each subgroup of age, sex, and social determinants of health as reference**

|  | Cardiovascular-kidney-metabolic syndrome stage^a^ | | | | | *P* for interaction^b^ |
| --- | --- | --- | --- | --- | --- | --- |
|  | 0 | 1 | 2 | 3 | 4 |  |
| **Education** |  |  |  |  |  | 0.812 |
| High school graduate or higher | 1.00 (Reference) | 0.53 (0.32-0.88) | 1.07 (0.67-1.69) | 1.48 (0.77-2.83) | 1.76 (1.03-3.00) |  |
| Less than high school | 1.00 (Reference) | 0.98 (0.71-1.36) | 1.35 (0.99-1.84) | 2.06 (1.29-3.27) | 2.19 (1.50-3.18) |  |
| **Marital status** |  |  |  |  |  | 0.461 |
| Married or living with a partner | 1.00 (Reference) | 0.69 (0.44-1.08) | 0.83 (0.56-1.21) | 1.71 (1.05-2.78) | 1.58 (1.02-2.47) |  |
| Not married nor living with a partner | 1.00 (Reference) | 1.12 (0.78-1.61) | 1.91 (1.37-2.67) | 2.09 (1.10-3.98) | 2.91 (1.92-4.41) |  |
| **Family income-to-poverty ratio** |  |  |  |  |  | 0.472 |
| ≥300% | 1.00 (Reference) | 0.67 (0.46-0.96) | 1.09 (0.78-1.51) | 1.84 (1.21-2.78) | 2.02 (1.41-2.90) |  |
| <300% | 1.00 (Reference) | 1.18 (0.76-1.85) | 1.62 (1.05-2.51) | 1.70 (0.72-4.01) | 2.08 (1.21-3.55) |  |
| **Food security** |  |  |  |  |  | 0.116 |
| Full security | 1.00 (Reference) | 0.92 (0.51-1.65) | 1.27 (0.73-2.21) | 2.25 (1.11-4.55) | 1.99 (1.13-3.51) |  |
| Marginal, low, or very low security | 1.00 (Reference) | 0.85 (0.61-1.18) | 1.30 (0.97-1.73) | 1.80 (1.11-2.92) | 2.24 (1.59-3.14) |  |
| **Type of health insurance** |  |  |  |  |  | 0.469 |
| Private | 1.00 (Reference) | 0.77 (0.47-1.26) | 1.34 (0.86-2.09) | 2.42 (1.46-4.01) | 2.44 (1.57-3.79) |  |
| Government or none | 1.00 (Reference) | 0.95 (0.66-1.36) | 1.30 (0.93-1.82) | 1.57 (0.85-2.90) | 1.93 (1.30-2.88) |  |
| **Employment status** |  |  |  |  |  | 0.597 |
| Employed, student, or retired | 1.00 (Reference) | 0.84 (0.51-1.38) | 0.95 (0.59-1.53) | 2.11 (1.00-4.42) | 1.82 (1.08-3.05) |  |
| Unemployed | 1.00 (Reference) | 0.89 (0.63-1.26) | 1.49 (1.05-2.11) | 1.83 (1.14-2.93) | 2.30 (1.57-3.39) |  |
| **Home ownership** |  |  |  |  |  | 0.297 |
| Own home | 1.00 (Reference) | 0.95 (0.57-1.56) | 1.35 (0.85-2.16) | 2.18 (1.16-4.09) | 2.46 (1.48-4.10) |  |
| Rent home or other arrangement | 1.00 (Reference) | 0.84 (0.61-1.17) | 1.27 (0.95-1.70) | 1.81 (1.11-2.94) | 2.01 (1.42-2.84) |  |
| **Regular health-care access** |  |  |  |  |  | 0.520 |
| At least one regular health-care facility | 1.00 (Reference) | 1.21 (0.65-2.23) | 1.38 (0.76-2.52) | 2.00 (0.77-5.22) | 2.04 (0.81-5.12) |  |
| None or emergency room | 1.00 (Reference) | 0.82 (0.60-1.13) | 1.29 (0.96-1.73) | 1.92 (1.26-2.94) | 2.14 (1.53-2.99) |  |
| **Cumulative unfavorable SDOHs** |  |  |  |  |  | 0.248 |
| <2 | 1.00 (Reference) | 0.77 (0.52-1.13) | 1.33 (0.93-1.90) | 2.23 (1.41-3.51) | 2.77 (1.92-3.98) |  |
| ≥2 | 1.00 (Reference) | 1.00 (0.66-1.53) | 1.53 (1.07-2.19) | 1.66 (0.88-3.13) | 2.44 (1.57-3.77) |  |

Abbreviation: SDOHs, social determinants of health.

^a^ All estimates accounted for complex survey designs. Models were adjusted for baseline age, sex, race/ethnicity, education, marital status, family income-to-poverty ratio, food security, type of health insurance, employment status, home ownership, regular health-care access, alcohol consumption, smoking status, physical activity, and medical history of cancer at baseline if applicable.

^b^ The multiplicative interaction between cardiovascular-kidney-metabolic syndrome stage and age, sex, and social determinants of health for the premature mortality were assessed by including their cross-product term in the model.

**eTable 6. Adjusted hazard ratios for risk of all-cause premature mortality according to cardiovascular-kidney-metabolic syndrome stages, with a subgroup of determinants of health as reference**

|  | Cardiovascular-kidney-metabolic syndrome stage^a^ | | | | |
| --- | --- | --- | --- | --- | --- |
|  | 0 | 1 | 2 | 3 | 4 |
| **Education** |  |  |  |  |  |
| High school graduate or higher | 1.00 (Reference) | 1.00 (Reference) | 1.00 (Reference) | 1.00 (Reference) | 1.00 (Reference) |
| Less than high school | 1.70 (0.90-3.23) | 1.00 (0.65-1.54) | 1.45 (1.15-1.82) | 1.04 (0.60-1.81) | 1.34 (0.97-1.84) |
| **Marital status** |  |  |  |  |  |
| Married or living with a partner | 1.00 (Reference) | 1.00 (Reference) | 1.00 (Reference) | 1.00 (Reference) | 1.00 (Reference) |
| Not married nor living with a partner | 2.60 (1.54-4.40) | 1.49 (1.03-2.16) | 1.15 (0.94-1.41) | 2.64 (1.54-4.53) | 1.45 (1.07-1.96) |
| **Family income-to-poverty ratio** |  |  |  |  |  |
| ≥300% | 1.00 (Reference) | 1.00 (Reference) | 1.00 (Reference) | 1.00 (Reference) | 1.00 (Reference) |
| <300% | 1.98 (0.91-4.33) | 0.98 (0.63-1.52) | 1.27 (0.97-1.67) | 1.53 (0.78-2.98) | 1.75 (1.14-2.67) |
| **Food security** |  |  |  |  |  |
| Full security | 1.00 (Reference) | 1.00 (Reference) | 1.00 (Reference) | 1.00 (Reference) | 1.00 (Reference) |
| Marginal, low, or very low security | 1.33 (0.65-2.73) | 1.67 (1.05-2.65) | 1.25 (1.01-1.54) | 1.13 (0.65-1.98) | 0.82 (0.57-1.19) |
| **Type of health insurance** |  |  |  |  |  |
| Private | 1.00 (Reference) | 1.00 (Reference) | 1.00 (Reference) | 1.00 (Reference) | 1.00 (Reference) |
| Government or none | 0.95 (0.48-1.89) | 1.03 (0.68-1.56) | 1.42 (1.08-1.87) | 1.45 (0.87-2.42) | 1.43 (1.03-1.98) |
| **Employment status** |  |  |  |  |  |
| Employed, student, or retired | 1.00 (Reference) | 1.00 (Reference) | 1.00 (Reference) | 1.00 (Reference) | 1.00 (Reference) |
| Unemployed | 2.41 (1.36-4.26) | 2.59 (1.70-3.93) | 1.45 (1.14-1.84) | 1.71 (0.84-3.48) | 1.72 (1.25-2.36) |
| **Home ownership** |  |  |  |  |  |
| Own home | 1.00 (Reference) | 1.00 (Reference) | 1.00 (Reference) | 1.00 (Reference) | 1.00 (Reference) |
| Rent home or other arrangement | 0.61 (0.35-1.06) | 0.98 (0.64-1.49) | 1.08 (0.88-1.32) | 0.96 (0.53-1.75) | 1.28 (0.92-1.80) |
| **Regular health-care access** |  |  |  |  |  |
| At least one regular health-care facility | 1.00 (Reference) | 1.00 (Reference) | 1.00 (Reference) | 1.00 (Reference) | 1.00 (Reference) |
| None or emergency room | 0.62 (0.31-1.23) | 1.15 (0.75-1.75) | 0.76 (0.54-1.07) | 0.80 (0.33-1.98) | 0.66 (0.30-1.42) |
| **Cumulative unfavorable SDOHs** |  |  |  |  |  |
| <2 | 1.00 (Reference) | 1.00 (Reference) | 1.00 (Reference) | 1.00 (Reference) | 1.00 (Reference) |
| ≥2 | 2.38 (1.37-4.14) | 1.73 (1.22-2.45) | 1.84 (1.53-2.21) | 2.93 (1.71-5.03) | 2.38 (1.78-3.19) |

Abbreviation: SDOHs, social determinants of health.

^a^ All estimates accounted for complex survey designs. Models were adjusted for baseline age, sex, race/ethnicity, education, marital status, family income-to-poverty ratio, food security, type of health insurance, employment status, home ownership, regular health-care access, alcohol consumption, smoking status, physical activity, and medical history of cancer at baseline if applicable.

**eTable 7. Adjusted hazard ratios for risk of all-cause premature mortality according to cardiovascular-kidney-metabolic syndrome stages by social determinants of health**

|  | Cardiovascular-kidney-metabolic syndrome stage^a^ | | | | |
| --- | --- | --- | --- | --- | --- |
|  | 0 | 1 | 2 | 3 | 4 |
| **Education** |  |  |  |  |  |
| High school graduate or higher | 1.00 (Reference) | 1.01 (0.73-1.39) | 1.42 (1.06-1.91) | 2.35 (1.53-3.62) | 2.39 (1.68-3.39) |
| Less than high school | 1.94 (1.19-3.18) | 1.05 (0.71-1.55) | 1.96 (1.37-2.79) | 2.39 (1.43-4.00) | 3.25 (2.23-4.72) |
| **Marital status** |  |  |  |  |  |
| Married or living with a partner | 1.00 (Reference) | 1.09 (0.76-1.56) | 1.83 (1.35-2.49) | 1.85 (1.03-3.31) | 2.79 (1.92-4.05) |
| Not married nor living with a partner | 2.41 (1.54-3.77) | 1.69 (1.13-2.53) | 2.08 (1.49-2.90) | 4.76 (3.06-7.39) | 4.07 (2.75-6.04) |
| **Family income-to-poverty ratio** |  |  |  |  |  |
| ≥300% | 1.00 (Reference) | 1.19 (0.76-1.86) | 1.67 (1.09-2.57) | 1.94 (0.89-4.21) | 2.37 (1.41-3.98) |
| <300% | 1.93 (1.17-3.18) | 1.30 (0.82-2.04) | 2.08 (1.27-3.38) | 3.44 (1.96-6.04) | 3.80 (2.33-6.19) |
| **Food security** |  |  |  |  |  |
| Full security | 1.00 (Reference) | 0.86 (0.62-1.19) | 1.35 (1.01-1.80) | 1.93 (1.22-3.05) | 2.38 (1.70-3.34) |
| Marginal, low, or very low security | 1.34 (0.77-2.35) | 1.23 (0.82-1.84) | 1.57 (1.09-2.26) | 2.55 (1.57-4.14) | 2.31 (1.53-3.49) |
| **Type of health insurance** |  |  |  |  |  |
| Private | 1.00 (Reference) | 0.97 (0.68-1.38) | 1.38 (1.00-1.92) | 1.82 (1.03-3.24) | 2.18 (1.47-3.22) |
| Government or none | 1.47 (0.87-2.48) | 1.11 (0.74-1.66) | 1.79 (1.20-2.67) | 2.93 (1.86-4.61) | 3.15 (2.08-4.76) |
| **Employment status** |  |  |  |  |  |
| Employed, student, or retired | 1.00 (Reference) | 0.89 (0.63-1.26) | 1.50 (1.07-2.11) | 1.88 (1.21-2.91) | 2.36 (1.62-3.42) |
| Unemployed | 2.34 (1.38-3.98) | 1.96 (1.31-2.96) | 2.17 (1.59-2.97) | 4.73 (2.53-8.82) | 4.16 (2.93-5.92) |
| **Home ownership** |  |  |  |  |  |
| Own home | 1.00 (Reference) | 0.83 (0.60-1.15) | 1.26 (0.95-1.66) | 1.75 (1.12-2.73) | 2.00 (1.44-2.77) |
| Rent home or other arrangement | 0.91 (0.59-1.41) | 0.88 (0.62-1.27) | 1.30 (0.96-1.75) | 2.21 (1.36-3.60) | 2.42 (1.70-3.43) |
| **Regular health-care access** |  |  |  |  |  |
| At least one regular health-care facility | 1.00 (Reference) | 0.82 (0.60-1.12) | 1.28 (0.96-1.70) | 1.89 (1.25-2.84) | 2.14 (1.56-2.95) |
| None or emergency room | 0.70 (0.38-1.30) | 0.85 (0.54-1.33) | 1.01 (0.67-1.52) | 1.55 (0.61-3.94) | 1.47 (0.67-3.24) |
| **Cumulative unfavorable SDOHs** |  |  |  |  |  |
| <2 | 1.00 (Reference) | 1.04 (0.68-1.58) | 1.63 (1.16-2.31) | 1.85 (1.03-3.32) | 2.65 (1.75-4.02) |
| ≥2 | 2.36 (1.47-3.80) | 1.78 (1.22-2.59) | 3.03 (2.06-4.46) | 4.93 (3.06-7.94) | 6.25 (4.20-9.32) |

Abbreviation: SDOHs, social determinants of health.

^a^ All estimates accounted for complex survey designs. Models were adjusted for baseline age, sex, race/ethnicity, education, marital status, family income-to-poverty ratio, food security, type of health insurance, employment status, home ownership, regular health-care access, alcohol consumption, smoking status, physical activity, and medical history of cancer at baseline if applicable.

^b^ Bolded data indicate among individuals at advanced cardiovascular-kidney-metabolic syndrome stages there were significant differences between those with favorable and unfavorable social determinants of health.

**eTable 8. Adjusted hazard ratios for risk of all-cause premature mortality according to cardiovascular-kidney-metabolic syndrome stages by social determinants of health among females and males, with stages 0-2 as reference**

| **CKM stage** | **Social determinants of health** | **Females** | | **Males** | | ***P* for sex interaction** |
| --- | --- | --- | --- | --- | --- | --- |
|  |  | Hazard ratio ^a^ | *P* values | Hazard ratio ^a^ | *P* values |  |
| **Categories** | **Education** |  |  |  |  | 0.365 |
| Stages 0-2 | High school graduate or higher | 1.00 (Reference) |  | 1.00 (Reference) |  |  |
| Stages 0-2 | Less than high school | 1.28 (0.94-1.74) | 0.116 | 1.46 (1.15-1.86) | 0.002 |  |
| Stages 3-4 | High school graduate or higher | 1.91 (1.35-2.69) | <0.001 | 1.81 (1.41-2.32) | <0.001 |  |
| Stages 3-4 | Less than high school | 3.02 (2.15-4.25) | <0.001 | 1.99 (1.49-2.67) | <0.001 |  |
| **Categories** | **Marital status** |  |  |  |  | 0.243 |
| Stages 0-2 | Married or living with a partner | 1.00 (Reference) |  | 1.00 (Reference) |  |  |
| Stages 0-2 | Not married nor living with a partner | 1.21 (0.91-1.60) | 0.186 | 1.44 (1.15-1.82) | 0.002 |  |
| Stages 3-4 | Married or living with a partner | 2.10 (1.46-3.01) | <0.001 | 1.47 (1.12-1.93) | 0.006 |  |
| Stages 3-4 | Not married nor living with a partner | 2.49 (1.75-3.55) | <0.001 | 2.90 (2.13-3.93) | <0.001 |  |
| **Categories** | **Family income-to-poverty ratio** |  |  |  |  | 0.556 |
| Stages 0-2 | ≥300% | 1.00 (Reference) |  | 1.00 (Reference) |  |  |
| Stages 0-2 | <300% | 1.34 (0.96-1.88) | 0.085 | 1.26 (0.98-1.63) | 0.068 |  |
| Stages 3-4 | ≥300% | 1.81 (1.06-3.11) | 0.031 | 1.42 (1.00-2.03) | 0.052 |  |
| Stages 3-4 | <300% | 2.93 (1.99-4.32) | <0.001 | 2.29 (1.68-3.11) | <0.001 |  |
| **Categories** | **Food security** |  |  |  |  | 0.091 |
| Stages 0-2 | Full security | 1.00 (Reference) |  | 1.00 (Reference) |  |  |
| Stages 0-2 | Marginal, low, or very low security | 1.43 (1.09-1.87) | 0.011 | 1.20 (0.96-1.49) | 0.104 |  |
| Stages 3-4 | Full security | 2.20 (1.59-3.03) | <0.001 | 1.76 (1.41-2.21) | <0.001 |  |
| Stages 3-4 | Marginal, low, or very low security | 2.66 (1.80-3.95) | <0.001 | 1.63 (1.16-2.30) | 0.005 |  |
| **Categories** | **Type of health insurance** |  |  |  |  | 0.579 |
| Stages 0-2 | Private | 1.00 (Reference) |  | 1.00 (Reference) |  |  |
| Stages 0-2 | Government or none | 1.13 (0.84-1.51) | 0.413 | 1.39 (1.05-1.84) | 0.021 |  |
| Stages 3-4 | Private | 2.14 (1.42-3.22) | <0.001 | 1.47 (1.09-1.99) | 0.011 |  |
| Stages 3-4 | Government or none | 2.29 (1.62-3.24) | <0.001 | 2.53 (1.88-3.40) | <0.001 |  |
| **Categories** | **Employment status** |  |  |  |  | 0.030 |
| Stages 0-2 | Employed, student, or retired | 1.00 (Reference) |  | 1.00 (Reference) |  |  |
| Stages 0-2 | Unemployed | 2.04 (1.58-2.65) | <0.001 | 1.39 (1.14-1.71) | 0.002 |  |
| Stages 3-4 | Employed, student, or retired | 2.10 (1.52-2.89) | <0.001 | 1.52 (1.17-1.98) | 0.002 |  |
| Stages 3-4 | Unemployed | 4.22 (2.98-5.97) | <0.001 | 2.72 (2.07-3.57) | <0.001 |  |
| **Categories** | **Home ownership** |  |  |  |  | 0.570 |
| Stages 0-2 | Own home | 1.00 (Reference) |  | 1.00 (Reference) |  |  |
| Stages 0-2 | Rent home or other arrangement | 0.98 (0.75-1.28) | 0.863 | 1.02 (0.82-1.28) | 0.839 |  |
| Stages 3-4 | Own home | 1.88 (1.30-2.72) | 0.001 | 1.59 (1.26-2.02) | <0.001 |  |
| Stages 3-4 | Rent home or other arrangement | 2.44 (1.77-3.35) | <0.001 | 1.84 (1.34-2.53) | <0.001 |  |
| **Categories** | **Regular health-care access** |  |  |  |  | 0.335 |
| Stages 0-2 | At least one regular health-care facility | 1.00 (Reference) |  | 1.00 (Reference) |  |  |
| Stages 0-2 | None or emergency room | 0.80 (0.50-1.28) | 0.351 | 0.80 (0.59-1.09) | 0.151 |  |
| Stages 3-4 | At least one regular health-care facility | 2.10 (1.62-2.72) | <0.001 | 1.65 (1.33-2.05) | <0.001 |  |
| Stages 3-4 | None or emergency room | 1.26 (0.42-3.74) | 0.675 | 1.28 (0.64-2.57) | 0.490 |  |
| **Categories** | **Cumulative unfavorable SDOHs** |  |  |  |  | 0.303 |
| Stages 0-2 | <2 | 1.00 (Reference) |  | 1.00 (Reference) |  |  |
| Stages 0-2 | ≥2 | 1.91 (1.47-2.48) | <0.001 | 1.89 (1.58-2.26) | <0.001 |  |
| Stages 3-4 | <2 | 1.92 (1.15-3.19) | 0.013 | 1.63 (1.22-2.16) | <0.001 |  |
| Stages 3-4 | ≥2 | 5.02 (3.63-6.95) | <0.001 | 3.69 (2.85-4.77) | <0.001 |  |

Abbreviation: SDOHs, social determinants of health.

^a^ All estimates accounted for complex survey designs. Models were adjusted for baseline age, race/ethnicity, education, marital status, family income-to-poverty ratio, food security, type of health insurance, employment status, home ownership, regular health-care access, alcohol consumption, smoking status, physical activity, and medical history of cancer at baseline if applicable. **eTable 9. Adjusted hazard ratios for risk of all-cause premature mortality according to cardiovascular-kidney-metabolic syndrome stages by social determinants of health among females and males, with stages 3-4 as reference**

| **CKM stage** | **Social determinants of health** | **Females** | | **Males** | | ***P* for sex interaction** |
| --- | --- | --- | --- | --- | --- | --- |
|  |  | Hazard ratio ^a^ | *P* values | Hazard ratio ^a^ | *P* values |  |
| **Categories** | **Education** |  |  |  |  | 0.365 |
| Stages 0-2 | High school graduate or higher | 0.52 (0.37-0.74) | <0.001 | 0.55 (0.43-0.71) | <0.001 |  |
| Stages 0-2 | Less than high school | 0.67 (0.46-0.97) | 0.033 | 0.81 (0.60-1.09) | 0.163 |  |
| Stages 3-4 | High school graduate or higher | 1.00 (Reference) |  | 1.00 (Reference) |  |  |
| Stages 3-4 | Less than high school | 1.58 (1.06-2.37) | 0.026 | 1.10 (0.79-1.53) | 0.559 |  |
| **Categories** | **Marital status** |  |  |  |  | 0.243 |
| Stages 0-2 | Married or living with a partner | 0.48 (0.33-0.69) | <0.001 | 0.68 (0.52-0.89) | 0.006 |  |
| Stages 0-2 | Not married nor living with a partner | 0.58 (0.40-0.83) | 0.004 | 0.98 (0.76-1.26) | 0.881 |  |
| Stages 3-4 | Married or living with a partner | 1.00 (Reference) |  | 1.00 (Reference) |  |  |
| Stages 3-4 | Not married nor living with a partner | 1.19 (0.79-1.78) | 0.402 | 1.97 (1.43-2.71) | <0.001 |  |
| **Categories** | **Family income-to-poverty ratio** |  |  |  |  | 0.556 |
| Stages 0-2 | ≥300% | 0.55 (0.32-0.95) | 0.031 | 0.70 (0.49-1.00) | 0.052 |  |
| Stages 0-2 | <300% | 0.74 (0.48-1.14) | 0.174 | 0.89 (0.62-1.28) | 0.528 |  |
| Stages 3-4 | ≥300% | 1.00 (Reference) |  | 1.00 (Reference) |  |  |
| Stages 3-4 | <300% | 1.62 (1.00-2.61) | 0.050 | 1.61 (1.12-2.31) | 0.010 |  |
| **Categories** | **Food security** |  |  |  |  | 0.091 |
| Stages 0-2 | Full security | 0.46 (0.33-0.63) | <0.001 | 0.57 (0.45-0.71) | <0.001 |  |
| Stages 0-2 | Marginal, low, or very low security | 0.65 (0.46-0.93) | 0.017 | 0.68 (0.51-0.91) | 0.010 |  |
| Stages 3-4 | Full security | 1.00 (Reference) |  | 1.00 (Reference) |  |  |
| Stages 3-4 | Marginal, low, or very low security | 1.21 (0.78-1.88) | 0.384 | 0.93 (0.66-1.30) | 0.652 |  |
| **Categories** | **Type of health insurance** |  |  |  |  | 0.579 |
| Stages 0-2 | Private | 0.47 (0.31-0.70) | <0.001 | 0.68 (0.50-0.91) | 0.011 |  |
| Stages 0-2 | Government or none | 0.53 (0.35-0.80) | 0.003 | 0.94 (0.70-1.28) | 0.711 |  |
| Stages 3-4 | Private | 1.00 (Reference) |  | 1.00 (Reference) |  |  |
| Stages 3-4 | Government or none | 1.07 (0.67-1.72) | 0.770 | 1.72 (1.28-2.30) | <0.001 |  |
| **Categories** | **Employment status** |  |  |  |  | 0.030 |
| Stages 0-2 | Employed, student, or retired | 0.48 (0.35-0.66) | <0.001 | 0.66 (0.51-0.86) | 0.002 |  |
| Stages 0-2 | Unemployed | 0.97 (0.67-1.43) | 0.896 | 0.92 (0.68-1.23) | 0.561 |  |
| Stages 3-4 | Employed, student, or retired | 1.00 (Reference) |  | 1.00 (Reference) |  |  |
| Stages 3-4 | Unemployed | 2.01 (1.30-3.11) | 0.002 | 1.79 (1.30-2.46) | <0.001 |  |
| **Categories** | **Home ownership** |  |  |  |  | 0.570 |
| Stages 0-2 | Own home | 0.53 (0.37-0.77) | 0.001 | 0.63 (0.50-0.79) | <0.001 |  |
| Stages 0-2 | Rent home or other arrangement | 0.52 (0.36-0.76) | <0.001 | 0.64 (0.47-0.88) | 0.0061 |  |
| Stages 3-4 | Own home | 1.00 (Reference) |  | 1.00 (Reference) |  |  |
| Stages 3-4 | Rent home or other arrangement | 1.30 (0.83-2.02) | 0.248 | 1.16 (0.82-1.63) | 0.402 |  |
| **Categories** | **Regular health-care access** |  |  |  |  | 0.335 |
| Stages 0-2 | At least one regular health-care facility | 0.48 (0.37-0.62) | <0.001 | 0.61 (0.49-0.75) | <0.001 |  |
| Stages 0-2 | None or emergency room | 0.38 (0.23-0.63) | <0.001 | 0.48 (0.34-0.69) | <0.001 |  |
| Stages 3-4 | At least one regular health-care facility | 1.00 (Reference) |  | 1.00 (Reference) |  |  |
| Stages 3-4 | None or emergency room | 0.60 (0.21-1.73) | 0.344 | 0.77 (0.38-1.57) | 0.475 |  |
| **Categories** | **Cumulative unfavorable SDOHs** |  |  |  |  | 0.303 |
| Stages 0-2 | <2 | 0.52 (0.31-0.87) | 0.013 | 0.61 (0.46-0.82) | <0.001 |  |
| Stages 0-2 | ≥2 | 1.00 (0.62-1.60) | 0.987 | 1.16 (0.90-1.50) | 0.252 |  |
| Stages 3-4 | <2 | 1.00 (Reference) |  | 1.00 (Reference) |  |  |
| Stages 3-4 | ≥2 | 2.62 (1.59-4.32) | <0.001 | 2.27 (1.73-2.98) | <0.001 |  |

Abbreviation: SDOHs, social determinants of health.

^a^ All estimates accounted for complex survey designs. Models were adjusted for baseline age, race/ethnicity, education, marital status, family income-to-poverty ratio, food security, type of health insurance, employment status, home ownership, regular health-care access, alcohol consumption, smoking status, physical activity, and medical history of cancer at baseline if applicable.

**eTable 10. Adjusted hazard ratios for risk of premature mortality from all causes according to cardiovascular-kidney-metabolic syndrome stages by social determinants of health across age groups, with stages 0-2 as reference**

| **CKM stage** | **Social determinants of health** | **Middle-aged** | | **Older** | | ***P* for age interaction** |
| --- | --- | --- | --- | --- | --- | --- |
|  |  | Hazard ratio^a^ | *P* values | Hazard ratio^a^ | *P* values |  |
| **Categories** | **Education** |  |  |  |  | 0.155 |
| Stages 0-2 | High school graduate or higher | 1.00 (Reference) |  | 1.00 (Reference) |  |  |
| Stages 0-2 | Less than high school | 1.46 (1.16-1.85) | 0.002 | 1.29 (0.97-1.71) | 0.078 |  |
| Stages 3-4 | High school graduate or higher | 2.11 (1.59-2.80) | <0.001 | 1.95 (1.50-2.52) | <0.001 |  |
| Stages 3-4 | Less than high school | 2.69 (1.86-3.88) | <0.001 | 2.56 (1.87-3.52) | <0.001 |  |
| **Categories** | **Marital status** |  |  |  |  | 0.310 |
| Stages 0-2 | Married or living with a partner | 1.00 (Reference) |  | 1.00 (Reference) |  |  |
| Stages 0-2 | Not married nor living with a partner | 1.18 (0.93-1.49) | 0.165 | 1.37 (1.04-1.81) | 0.025 |  |
| Stages 3-4 | Married or living with a partner | 1.89 (1.39-2.58) | <0.001 | 1.83 (1.38-2.44) | <0.001 |  |
| Stages 3-4 | Not married nor living with a partner | 2.56 (1.80-3.65) | <0.001 | 2.95 (2.15-4.04) | <0.001 |  |
| **Categories** | **Family income-to-poverty ratio** |  |  |  |  | 0.905 |
| Stages 0-2 | ≥300% | 1.00 (Reference) |  | 1.00 (Reference) |  |  |
| Stages 0-2 | <300% | 1.28 (0.98-1.66) | 0.069 | 1.20 (0.90-1.61) | 0.219 |  |
| Stages 3-4 | ≥300% | 2.14 (1.39-3.28) | 0.001 | 1.40 (0.94-2.08) | 0.093 |  |
| Stages 3-4 | <300% | 2.47 (1.79-3.40) | <0.001 | 2.82 (1.96-4.05) | <0.001 |  |
| **Categories** | **Food security** |  |  |  |  | 0.377 |
| Stages 0-2 | Full security | 1.00 (Reference) |  | 1.00 (Reference) |  |  |
| Stages 0-2 | Marginal, low, or very low security | 1.36 (1.08-1.70) | 0.008 | 0.94 (0.67-1.31) | 0.697 |  |
| Stages 3-4 | Full security | 2.33 (1.73-3.15) | <0.001 | 1.89 (1.51-2.37) | <0.001 |  |
| Stages 3-4 | Marginal, low, or very low security | 2.10 (1.49-2.98) | <0.001 | 2.12 (1.48-3.05) | <0.001 |  |
| **Categories** | **Type of health insurance** |  |  |  |  | 0.133 |
| Stages 0-2 | Private | 1.00 (Reference) |  | 1.00 (Reference) |  |  |
| Stages 0-2 | Government or none | 1.53 (1.14-2.04) | 0.004 | 1.08 (0.83-1.39) | 0.575 |  |
| Stages 3-4 | Private | 1.85 (1.25-2.72) | 0.002 | 1.77 (1.32-2.37) | 0.000 |  |
| Stages 3-4 | Government or none | 3.24 (2.24-4.70) | <0.001 | 2.35 (1.81-3.04) | <0.001 |  |
| **Categories** | **Employment status** |  |  |  |  | 0.197 |
| Stages 0-2 | Employed, student, or retired | 1.00 (Reference) |  | 1.00 (Reference) |  |  |
| Stages 0-2 | Unemployed | 1.64 (1.34-2.00) | <0.001 | 1.28 (0.94-1.73) | 0.113 |  |
| Stages 3-4 | Employed, student, or retired | 1.99 (1.44-2.76) | <0.001 | 1.83 (1.41-2.38) | <0.001 |  |
| Stages 3-4 | Unemployed | 3.29 (2.41-4.50) | <0.001 | 3.00 (2.23-4.03) | <0.001 |  |
| **Categories** | **Home ownership** |  |  |  |  | 0.199 |
| Stages 0-2 | Own home | 1.00 (Reference) |  | 1.00 (Reference) |  |  |
| Stages 0-2 | Rent home or other arrangement | 0.95 (0.79-1.15) | 0.618 | 1.33 (0.90-1.97) | 0.153 |  |
| Stages 3-4 | Own home | 1.86 (1.34-2.57) | <0.001 | 1.97 (1.53-2.54) | <0.001 |  |
| Stages 3-4 | Rent home or other arrangement | 2.14 (1.64-2.80) | <0.001 | 2.57 (1.81-3.63) | <0.001 |  |
| **Categories** | **Regular health-care access** |  |  |  |  | 0.121 |
| Stages 0-2 | At least one regular health-care facility | 1.00 (Reference) |  | 1.00 (Reference) |  |  |
| Stages 0-2 | None or emergency room | 0.89 (0.66-1.18) | 0.406 | 0.75 (0.46-1.23) | 0.251 |  |
| Stages 3-4 | At least one regular health-care facility | 2.05 (1.63-2.57) | <0.001 | 1.95 (1.57-2.42) | <0.001 |  |
| Stages 3-4 | None or emergency room | 1.26 (0.57-2.78) | 0.561 | 1.66 (0.76-3.65) | 0.202 |  |
| **Categories** | **Cumulative unfavorable SDOHs** |  |  |  |  | 0.311 |
| Stages 0-2 | <2 | 1.00 (Reference) |  | 1.00 (Reference) |  |  |
| Stages 0-2 | ≥2 | 2.03 (1.65-2.49) | <0.001 | 1.57 (1.23-2.01) | <0.001 |  |
| Stages 3-4 | <2 | 2.48 (1.71-3.60) | <0.001 | 1.54 (1.14-2.09) | 0.006 |  |
| Stages 3-4 | ≥2 | 4.88 (3.66-6.53) | <0.001 | 3.99 (2.97-5.38) | <0.001 |  |

Abbreviation: SDOHs, social determinants of health.

^a^ All estimates accounted for complex survey designs. Models were adjusted for sex, race/ethnicity, education, marital status, family income-to-poverty ratio, food security, type of health insurance, employment status, home ownership, regular health-care access, alcohol consumption, smoking status, physical activity, and medical history of cancer at baseline if applicable.

**eTable 11. Adjusted hazard ratios for risk of premature mortality from all causes according to cardiovascular-kidney-metabolic syndrome stages by social determinants of health across age groups, with stages 3-4 as reference**

| **CKM stage** | **Social determinants of health** | **Middle-aged** | | **Older** | | ***P* for age interaction** |
| --- | --- | --- | --- | --- | --- | --- |
|  |  | Hazard ratio^a^ | *P* values | Hazard ratio^a^ | *P* values |  |
| **Categories** | **Education** |  |  |  |  | 0.155 |
| Stages 0-2 | High school graduate or higher | 0.47 (0.36-0.63) | <0.001 | 0.51 (0.40-0.67) | <0.001 |  |
| Stages 0-2 | Less than high school | 0.69 (0.51-0.94) | 0.018 | 0.66 (0.48-0.90) | 0.010 |  |
| Stages 3-4 | High school graduate or higher | 1.00 (Reference) |  | 1.00 (Reference) |  |  |
| Stages 3-4 | Less than high school | 1.28 (0.80-2.03) | 0.302 | 1.32 (0.96-1.81) | 0.089 |  |
| **Categories** | **Marital status** |  |  |  |  | 0.310 |
| Stages 0-2 | Married or living with a partner | 0.53 (0.39-0.72) | <0.001 | 0.55 (0.41-0.73) | <0.001 |  |
| Stages 0-2 | Not married nor living with a partner | 0.62 (0.46-0.85) | 0.003 | 0.75 (0.56-1.00) | 0.048 |  |
| Stages 3-4 | Married or living with a partner | 1.00 (Reference) |  | 1.00 (Reference) |  |  |
| Stages 3-4 | Not married nor living with a partner | 1.35 (0.87-2.11) | 0.178 | 1.61 (1.19-2.17) | 0.002 |  |
| **Categories** | **Family income-to-poverty ratio** |  |  |  |  | 0.905 |
| Stages 0-2 | ≥300% | 0.47 (0.30-0.72) | 0.001 | 0.71 (0.48-1.06) | 0.093 |  |
| Stages 0-2 | <300% | 0.60 (0.39-0.93) | 0.022 | 0.86 (0.58-1.27) | 0.438 |  |
| Stages 3-4 | ≥300% | 1.00 (Reference) |  | 1.00 (Reference) |  |  |
| Stages 3-4 | <300% | 1.15 (0.70-1.89) | 0.566 | 2.01 (1.35-3.00) | 0.001 |  |
| **Categories** | **Food security** |  |  |  |  | 0.377 |
| Stages 0-2 | Full security | 0.43 (0.32-0.58) | <0.001 | 0.53 (0.42-0.66) | <0.001 |  |
| Stages 0-2 | Marginal, low, or very low security | 0.58 (0.43-0.79) | 0.001 | 0.49 (0.34-0.71) | <0.001 |  |
| Stages 3-4 | Full security | 1.00 (Reference) |  | 1.00 (Reference) |  |  |
| Stages 3-4 | Marginal, low, or very low security | 0.90 (0.58-1.40) | 0.642 | 1.12 (0.80-1.57) | 0.498 |  |
| **Categories** | **Type of health insurance** |  |  |  |  | 0.133 |
| Stages 0-2 | Private | 0.54 (0.37-0.80) | 0.002 | 0.57 (0.42-0.76) | <0.001 |  |
| Stages 0-2 | Government or none | 0.83 (0.58-1.19) | 0.306 | 0.61 (0.44-0.85) | 0.003 |  |
| Stages 3-4 | Private | 1.00 (Reference) |  | 1.00 (Reference) |  |  |
| Stages 3-4 | Government or none | 1.75 (1.11-2.78) | 0.017 | 1.33 (1.01-1.74) | 0.043 |  |
| **Categories** | **Employment status** |  |  |  |  | 0.197 |
| Stages 0-2 | Employed, student, or retired | 0.50 (0.36-0.70) | <0.001 | 0.55 (0.42-0.71) | <0.001 |  |
| Stages 0-2 | Unemployed | 0.82 (0.56-1.21) | 0.318 | 0.70 (0.49-1.00) | 0.048 |  |
| Stages 3-4 | Employed, student, or retired | 1.00 (Reference) |  | 1.00 (Reference) |  |  |
| Stages 3-4 | Unemployed | 1.66 (1.03-2.65) | 0.036 | 1.64 (1.16-2.32) | 0.006 |  |
| **Categories** | **Home ownership** |  |  |  |  | 0.199 |
| Stages 0-2 | Own home | 0.54 (0.39-0.75) | <0.001 | 0.51 (0.39-0.66) | <0.001 |  |
| Stages 0-2 | Rent home or other arrangement | 0.51 (0.37-0.72) | <0.001 | 0.68 (0.46-1.00) | 0.050 |  |
| Stages 3-4 | Own home | 1.00 (Reference) |  | 1.00 (Reference) |  |  |
| Stages 3-4 | Rent home or other arrangement | 1.15 (0.76-1.74) | 0.497 | 1.30 (0.92-1.86) | 0.138 |  |
| **Categories** | **Regular health-care access** |  |  |  |  | 0.121 |
| Stages 0-2 | At least one regular health-care facility | 0.49 (0.39-0.61) | <0.001 | 0.51 (0.41-0.64) | <0.001 |  |
| Stages 0-2 | None or emergency room | 0.43 (0.30-0.61) | <0.001 | 0.38 (0.23-0.63) | <0.001 |  |
| Stages 3-4 | At least one regular health-care facility | 1.00 (Reference) |  | 1.00 (Reference) |  |  |
| Stages 3-4 | None or emergency room | 0.62 (0.27-1.42) | 0.254 | 0.85 (0.40-1.84) | 0.682 |  |
| **Categories** | **Cumulative unfavorable SDOHs** |  |  |  |  | 0.311 |
| Stages 0-2 | <2 | 0.40 (0.28-0.58) | <0.001 | 0.65 (0.48-0.88) | 0.006 |  |
| Stages 0-2 | ≥2 | 0.82 (0.56-1.19) | 0.288 | 1.02 (0.76-1.37) | 0.904 |  |
| Stages 3-4 | <2 | 1.00 (Reference) |  | 1.00 (Reference) |  |  |
| Stages 3-4 | ≥2 | 1.97 (1.29-3.00) | 0.002 | 2.59 (1.92-3.48) | <0.001 |  |

Abbreviation: SDOHs, social determinants of health.

^a^ All estimates accounted for complex survey designs. Models were adjusted for sex, race/ethnicity, education, marital status, family income-to-poverty ratio, food security, type of health insurance, employment status, home ownership, regular health-care access, alcohol consumption, smoking status, physical activity, and medical history of cancer at baseline if applicable.

**eTable 12. Adjusted hazard ratios for risk of premature mortality from cardiovascular disease according to cardiovascular-kidney-metabolic syndrome stages, with stage 0 in each subgroup of age, sex, and social determinants of health as reference**

|  | Cardiovascular-kidney-metabolic syndrome stage^a^ | | | | | *P* for interaction^b^ |
| --- | --- | --- | --- | --- | --- | --- |
|  | 0 | 1 | 2 | 3 | 4 |  |
| **Education** |  |  |  |  |  | 0.182 |
| High school graduate or higher | 1.00 (Reference) | 1.27 (0.49-3.34) | 1.44 (0.52-3.95) | 3.44 (1.11-10.66) | 5.72 (2.26-14.45) |  |
| Less than high school | 1.00 (Reference) | 0.26 (0.08-0.90) | 3.01 (1.18-7.70) | 5.60 (1.48-21.13) | 8.63 (3.19-23.37) |  |
| **Marital status** |  |  |  |  |  | 0.829 |
| Married or living with a partner | 1.00 (Reference) | 1.05 (0.32-3.47) | 1.60 (0.48-5.36) | 2.52 (0.59-10.74) | 5.86 (1.90-18.06) |  |
| Not married nor living with a partner | 1.00 (Reference) | 1.19 (0.41-3.47) | 1.86 (0.75-4.64) | 5.83 (1.82-18.68) | 6.91 (2.96-16.12) |  |
| **Family income-to-poverty ratio** |  |  |  |  |  | 0.911 |
| ≥300% | 1.00 (Reference) | 1.38 (0.37-5.24) | 1.44 (0.32-6.43) | 1.93 (0.33-11.45) | 6.20 (1.59-24.19) |  |
| <300% | 1.00 (Reference) | 0.87 (0.32-2.41) | 1.82 (0.78-4.27) | 4.41 (1.60-12.11) | 6.33 (2.79-14.36) |  |
| **Food security** |  |  |  |  |  | 0.092 |
| Full security | 1.00 (Reference) | 1.26 (0.47-3.40) | 1.73 (0.62-4.77) | 4.07 (1.33-12.48) | 7.59 (3.20-18.01) |  |
| Marginal, low, or very low security | 1.00 (Reference) | 0.71 (0.16-3.18) | 1.56 (0.47-5.21) | 3.07 (0.82-11.53) | 4.11 (1.34-12.61) |  |
| **Type of health insurance** |  |  |  |  |  | 0.145 |
| Private | 1.00 (Reference) | 1.02 (0.33-3.14) | 1.40 (0.40-4.89) | 2.45 (0.67-9.03) | 6.64 (2.17-20.31) |  |
| Government or none | 1.00 (Reference) | 1.18 (0.38-3.71) | 2.10 (0.83-5.26) | 5.36 (1.65-17.42) | 6.26 (2.47-15.86) |  |
| **Employment status** |  |  |  |  |  | 0.715 |
| Employed, student, or retired | 1.00 (Reference) | 1.09 (0.46-2.61) | 1.47 (0.62-3.48) | 1.75 (0.78-3.92) | 5.65 (2.63-12.17) |  |
| Unemployed | 1.00 (Reference) | 1.15 (0.15-8.71) | 3.01 (0.44-20.68) | 18.94 (2.01-178.6) | 10.70 (1.51-75.83) |  |
| **Home ownership** |  |  |  |  |  | 0.857 |
| Own home | 1.00 (Reference) | 0.76 (0.29-2.03) | 1.05 (0.40-2.74) | 1.96 (0.69-5.55) | 4.02 (1.62-9.95) |  |
| Rent home or other arrangement | 1.00 (Reference) | 2.56 (0.51-12.81) | 4.98 (1.16-21.32) | 14.87 (2.82-78.41) | 16.36 (3.89-68.85) |  |
| **Regular health-care access** |  |  |  |  |  | 0.065 |
| At least one regular health-care facility | 1.00 (Reference) | 0.91 (0.35-2.35) | 1.66 (0.66-4.21) | 3.56 (1.33-9.52) | 6.14 (2.75-13.72) |  |
| None or emergency room | 1.00 (Reference) | 2.36 (0.60-9.19) | 1.95 (0.46-8.32) | 10.54 (1.52-73.21) | 3.18 (0.57-17.71) |  |
| **Cumulative unfavorable SDOHs** |  |  |  |  |  | 0.695 |
| <2 | 1.00 (Reference) | 1.13 (0.44-2.91) | 2.39 (1.05-5.44) | 6.23 (2.23-17.41) | 8.77 (3.97-19.4) |  |
| ≥2 | 1.00 (Reference) | 0.74 (0.25-2.19) | 0.89 (0.31-2.50) | 0.80 (0.25-2.58) | 3.68 (1.33-10.2) |  |

Abbreviation: SDOHs, social determinants of health.

^a^ All estimates accounted for complex survey designs. Models were adjusted for baseline age, sex, race/ethnicity, education, marital status, family income-to-poverty ratio, food security, type of health insurance, employment status, home ownership, regular health-care access, alcohol consumption, smoking status, physical activity, and medical history of cancer at baseline if applicable.

^b^ The multiplicative interaction between cardiovascular-kidney-metabolic syndrome stage and age, sex, and social determinants of health for the premature mortality were assessed by including their cross-product term in the model.

**eTable 13. Adjusted hazard ratios for risk of premature mortality from cardiovascular disease according to cardiovascular-kidney-metabolic syndrome stages by social determinants of health: Fine and Gray Cox regression models**

| **CKM stage** | **Social determinants of health** | **Hazard ratio^a^** | ***P* values** |  | **Hazard ratio^a^** | ***P* values** |
| --- | --- | --- | --- | --- | --- | --- |
| **Categories** | **Education** |  |  |  |  |  |
| Stages 0-2 | High school graduate or higher | 1.00 (Reference) |  |  | 0.54 (0.45-0.66) | <0.001 |
| Stages 0-2 | Less than high school | 1.39 (1.15-1.67) | 0.001 |  | 0.75 (0.61-0.94) | 0.011 |
| Stages 3-4 | High school graduate or higher | 1.84 (1.51-2.24) | <0.001 |  | 1.00 (Reference) |  |
| Stages 3-4 | Less than high school | 2.35 (1.84-3.01) | <0.001 |  | 1.28 (0.97-1.68) | 0.078 |
| **Categories** | **Marital status** |  |  |  |  |  |
| Stages 0-2 | Married or living with a partner | 1.00 (Reference) |  |  | 0.61 (0.49-0.75) | <0.001 |
| Stages 0-2 | Not married nor living with a partner | 1.33 (1.12-1.57) | 0.001 |  | 0.81 (0.65-1.00) | 0.049 |
| Stages 3-4 | Married or living with a partner | 1.65 (1.33-2.04) | <0.001 |  | 1.00 (Reference) |  |
| Stages 3-4 | Not married nor living with a partner | 2.67 (2.16-3.31) | <0.001 |  | 1.62 (1.27-2.08) | <0.001 |
| **Categories** | **Family income-to-poverty ratio** |  |  |  |  |  |
| Stages 0-2 | ≥300% | 1.00 (Reference) |  |  | 0.65 (0.49-0.87) | 0.004 |
| Stages 0-2 | <300% | 1.28 (1.04-1.56) | 0.019 |  | 0.83 (0.62-1.10) | 0.199 |
| Stages 3-4 | ≥300% | 1.54 (1.15-2.05) | 0.004 |  | 1.00 (Reference) |  |
| Stages 3-4 | <300% | 2.47 (1.93-3.16) | <0.001 |  | 1.61 (1.18-2.19) | 0.003 |
| **Categories** | **Food security** |  |  |  |  |  |
| Stages 0-2 | Full security | 1.00 (Reference) |  |  | 0.53 (0.44-0.63) | <0.001 |
| Stages 0-2 | Marginal, low, or very low security | 1.26 (1.06-1.50) | 0.009 |  | 0.67 (0.54-0.83) | 0.000 |
| Stages 3-4 | Full security | 1.89 (1.59-2.26) | <0.001 |  | 1.00 (Reference) |  |
| Stages 3-4 | Marginal, low, or very low security | 1.96 (1.51-2.55) | <0.001 |  | 1.04 (0.80-1.35) | 0.786 |
| **Categories** | **Type of health insurance** |  |  |  |  |  |
| Stages 0-2 | Private | 1.00 (Reference) |  |  | 0.60 (0.47-0.77) | <0.001 |
| Stages 0-2 | Government or none | 1.28 (1.04-1.59) | 0.023 |  | 0.77 (0.61-0.98) | 0.034 |
| Stages 3-4 | Private | 1.66 (1.30-2.13) | <0.001 |  | 1.00 (Reference) |  |
| Stages 3-4 | Government or none | 2.44 (1.94-3.07) | <0.001 |  | 1.46 (1.15-1.87) | 0.002 |
| **Categories** | **Employment status** |  |  |  |  |  |
| Stages 0-2 | Employed, student, or retired | 1.00 (Reference) |  |  | 0.59 (0.48-0.73) | <0.001 |
| Stages 0-2 | Unemployed | 1.70 (1.44-2.01) | <0.001 |  | 1.00 (0.79-1.26) | 0.998 |
| Stages 3-4 | Employed, student, or retired | 1.70 (1.37-2.10) | <0.001 |  | 1.00 (Reference) |  |
| Stages 3-4 | Unemployed | 3.27 (2.62-4.09) | <0.001 |  | 1.92 (1.46-2.54) | <0.001 |
| **Categories** | **Home ownership** |  |  |  |  |  |
| Stages 0-2 | Own home | 1.00 (Reference) |  |  | 0.59 (0.49-0.72) | <0.001 |
| Stages 0-2 | Rent home or other arrangement | 1.01 (0.86-1.19) | 0.913 |  | 0.60 (0.47-0.76) | <0.001 |
| Stages 3-4 | Own home | 1.69 (1.39-2.06) | <0.001 |  | 1.00 (Reference) |  |
| Stages 3-4 | Rent home or other arrangement | 2.05 (1.68-2.51) | <0.001 |  | 1.22 (0.93-1.58) | 0.148 |
| **Categories** | **Regular health-care access** |  |  |  |  |  |
| Stages 0-2 | At least one regular health-care facility | 1.00 (Reference) |  |  | 0.56 (0.47-0.65) | <0.001 |
| Stages 0-2 | None or emergency room | 0.81 (0.64-1.02) | 0.072 |  | 0.45 (0.34-0.59) | <0.001 |
| Stages 3-4 | At least one regular health-care facility | 1.80 (1.53-2.11) | <0.001 |  | 1.00 (Reference) |  |
| Stages 3-4 | None or emergency room | 1.30 (0.74-2.30) | 0.360 |  | 0.72 (0.41-1.28) | 0.266 |
| **Categories** | **Cumulative unfavorable SDOHs** |  |  |  |  |  |
| Stages 0-2 | <2 | 1.00 (Reference) |  |  | 0.58 (0.45-0.74) | <0.001 |
| Stages 0-2 | ≥2 | 1.88 (1.60-2.22) | <0.001 |  | 1.09 (0.86-1.37) | 0.485 |
| Stages 3-4 | <2 | 1.73 (1.36-2.21) | <0.001 |  | 1.00 (Reference) |  |
| Stages 3-4 | ≥2 | 4.16 (3.35-5.18) | <0.001 |  | 2.40 (1.86-3.10) | <0.001 |

^a^ All estimates accounted for complex survey designs. Models were adjusted for baseline age, sex, race/ethnicity, education, marital status, family income-to-poverty ratio, food security, type of health insurance, employment status, home ownership, regular health-care access, alcohol consumption, smoking status, physical activity, and medical history of cancer at baseline if applicable.

**eTable 14. Adjusted hazard ratios for risk of premature mortality from cardiovascular disease according to cardiovascular-kidney-metabolic syndrome stages by social determinants of health**

|  | Cardiovascular-kidney-metabolic syndrome stage^a^ | | | | |
| --- | --- | --- | --- | --- | --- |
|  | 0 | 1 | 2 | 3 | 4 |
| **Education** |  |  |  |  |  |
| High school graduate or higher | 1.00 (Reference) | 1.26 (0.49-3.26) | 1.42 (0.53-3.80) | 3.57 (1.24-10.26) | 5.81 (2.44-13.79) |
| Less than high school | 0.77 (0.27-2.16) | 0.22 (0.07-0.71) | 2.64 (0.97-7.20) | 4.86 (1.40-16.85) | 8.19 (3.30-20.33) |
| **Marital status** |  |  |  |  |  |
| Married or living with a partner | 1.00 (Reference) | 1.05 (0.32-3.49) | 1.66 (0.50-5.52) | 2.64 (0.64-10.83) | 6.45 (2.19-18.97) |
| Not married nor living with a partner | 1.45 (0.42-5.00) | 1.76 (0.49-6.36) | 2.67 (0.81-8.80) | 7.94 (2.38-26.50) | 9.39 (3.05-28.91) |
| **Family income-to-poverty ratio** |  |  |  |  |  |
| ≥300% | 1.00 (Reference) | 1.48 (0.39-5.58) | 1.63 (0.37-7.18) | 3.39 (0.67-17.14) | 7.70 (2.15-27.52) |
| <300% | 1.78 (0.52-6.11) | 1.57 (0.43-5.80) | 3.17 (0.88-11.35) | 7.10 (1.77-28.46) | 10.48 (3.15-34.92) |
| **Food security** |  |  |  |  |  |
| Full security | 1.00 (Reference) | 1.27 (0.47-3.45) | 1.77 (0.63-4.95) | 4.32 (1.44-13.01) | 8.17 (3.43-19.49) |
| Marginal, low, or very low security | 1.98 (0.64-6.10) | 1.54 (0.39-6.10) | 3.18 (1.12-9.04) | 6.15 (1.84-20.56) | 7.50 (2.82-19.97) |
| **Type of health insurance** |  |  |  |  |  |
| Private | 1.00 (Reference) | 1.06 (0.34-3.30) | 1.54 (0.45-5.33) | 3.33 (0.93-11.88) | 8.05 (2.75-23.56) |
| Government or none | 1.40 (0.47-4.22) | 1.67 (0.48-5.76) | 2.73 (0.88-8.50) | 6.03 (1.68-21.60) | 7.56 (2.63-21.72) |
| **Employment status** |  |  |  |  |  |
| Employed, student, or retired | 1.00 (Reference) | 1.12 (0.47-2.66) | 1.59 (0.68-3.73) | 2.01 (0.91-4.46) | 6.46 (3.10-13.46) |
| Unemployed | 0.90 (0.18-4.66) | 1.02 (0.35-2.93) | 2.24 (1.00-5.00) | 11.54 (3.75-35.54) | 7.21 (3.73-13.92) |
| **Home ownership** |  |  |  |  |  |
| Own home | 1.00 (Reference) | 0.79 (0.30-2.07) | 1.15 (0.45-2.93) | 2.51 (0.93-6.75) | 4.95 (2.14-11.48) |
| Rent home or other arrangement | 0.38 (0.10-1.53) | 1.03 (0.33-3.15) | 1.78 (0.68-4.66) | 4.14 (1.23-13.88) | 5.00 (2.22-11.26) |
| **Regular health-care access** |  |  |  |  |  |
| At least one regular health-care facility | 1.00 (Reference) | 0.91 (0.35-2.33) | 1.66 (0.66-4.20) | 3.57 (1.33-9.57) | 6.25 (2.79-14.01) |
| None or emergency room | 0.54 (0.13-2.23) | 1.36 (0.47-3.95) | 1.09 (0.38-3.10) | 4.70 (1.24-17.79) | 1.68 (0.42-6.75) |
| **Cumulative unfavorable SDOHs** |  |  |  |  |  |
| <2 | 1.00 (Reference) | 0.82 (0.28-2.42) | 1.08 (0.39-2.99) | 1.32 (0.41-4.27) | 5.16 (2.02-13.2) |
| ≥2 | 1.55 (0.59-4.10) | 1.71 (0.62-4.69) | 3.41 (1.32-8.76) | 7.78 (2.50-24.2) | 11.9 (4.85-29.1) |

Abbreviation: SDOHs, social determinants of health.

^a^ All estimates accounted for complex survey designs. Models were adjusted for baseline age, sex, race/ethnicity, education, marital status, family income-to-poverty ratio, food security, type of health insurance, employment status, home ownership, regular health-care access, alcohol consumption, smoking status, physical activity, and medical history of cancer at baseline if applicable.

**eTable 15. Adjusted hazard ratios for risk of premature mortality from cardiovascular disease according to cardiovascular-kidney-metabolic syndrome, with a subgroup of social determinants of health as reference**

|  | Cardiovascular-kidney-metabolic syndrome stage^a^ | | | | |
| --- | --- | --- | --- | --- | --- |
|  | 0 | 1 | 2 | 3 | 4 |
| **Education** |  |  |  |  |  |
| High school graduate or higher | 1.00 (Reference) | 1.00 (Reference) | 1.00 (Reference) | 1.00 (Reference) | 1.00 (Reference) |
| Less than high school | 1.60 (0.55-4.65) | 1.60 (0.64-3.98) | 1.46 (0.93-2.29) | 2.28 (0.64-8.14) | 1.62 (1.01-2.59) |
| **Marital status** |  |  |  |  |  |
| Married or living with a partner | 1.00 (Reference) | 1.00 (Reference) | 1.00 (Reference) | 1.00 (Reference) | 1.00 (Reference) |
| Not married nor living with a partner | 2.37 (0.68-8.26) | 1.37 (0.44-4.26) | 1.55 (0.82-2.92) | 1.17 (0.23-6.01) | 1.81 (0.99-3.30) |
| **Family income-to-poverty ratio** |  |  |  |  |  |
| ≥300% | 1.00 (Reference) | 1.00 (Reference) | 1.00 (Reference) | 1.00 (Reference) | 1.00 (Reference) |
| <300% | 4.33 (1.55-12.09) | 1.51 (0.45-5.08) | 1.58 (0.90-2.78) | 1.34 (0.27-6.73) | 0.94 (0.55-1.62) |
| **Food security** |  |  |  |  |  |
| Full security | 1.00 (Reference) | 1.00 (Reference) | 1.00 (Reference) | 1.00 (Reference) | 1.00 (Reference) |
| Marginal, low, or very low security | 1.74 (0.51-5.86) | 1.94 (0.80-4.71) | 1.60 (0.96-2.67) | 1.26 (0.44-3.58) | 0.96 (0.53-1.77) |
| **Type of health insurance** |  |  |  |  |  |
| Private | 1.00 (Reference) | 1.00 (Reference) | 1.00 (Reference) | 1.00 (Reference) | 1.00 (Reference) |
| Government or none | 0.91 (0.09-9.01) | 1.17 (0.42-3.29) | 1.16 (0.76-1.77) | 3.56 (0.79-16.00) | 1.19 (0.76-1.85) |
| **Employment status** |  |  |  |  |  |
| Employed, student, or retired | 1.00 (Reference) | 1.00 (Reference) | 1.00 (Reference) | 1.00 (Reference) | 1.00 (Reference) |
| Unemployed | 0.23 (0.03-1.54) | 1.63 (0.59-4.48) | 1.33 (0.79-2.26) | 1.56 (0.58-4.20) | 1.20 (0.73-1.97) |
| **Home ownership** |  |  |  |  |  |
| Own home | 1.00 (Reference) | 1.00 (Reference) | 1.00 (Reference) | 1.00 (Reference) | 1.00 (Reference) |
| Rent home or other arrangement | 0.69 (0.16-2.93) | 1.53 (0.58-3.99) | 0.56 (0.27-1.16) | 3.14 (0.69-14.19) | 0.28 (0.08-1.03) |
| **Regular health-care access** |  |  |  |  |  |
| At least one regular health-care facility | 1.00 (Reference) | 1.00 (Reference) | 1.00 (Reference) | 1.00 (Reference) | 1.00 (Reference) |
| None or emergency room | 2.70 (0.90-8.09) | 5.05 (1.64-15.57) | 3.95 (2.43-6.44) | 3.23 (1.21-8.57) | 2.38 (1.42-3.99) |
| **Cumulative unfavorable SDOHs** |  |  |  |  |  |
| <2 | 1.00 (Reference) | 1.00 (Reference) | 1.00 (Reference) | 1.00 (Reference) | 1.00 (Reference) |
| ≥2 | 1.92 (0.73-5.04) | 2.79 (1.06-7.36) | 2.89 (1.92-4.35) | 4.66 (1.31-16.52) | 2.20 (1.38-3.51) |

Abbreviation: SDOHs, social determinants of health.

^a^ All estimates accounted for complex survey designs. Models were adjusted for baseline age, sex, race/ethnicity, education, marital status, family income-to-poverty ratio, food security, type of health insurance, employment status, home ownership, regular health-care access, alcohol consumption, smoking status, physical activity, and medical history of cancer at baseline if applicable.

**eTable 16. Adjusted hazard ratios for risk of premature mortality from cardiovascular disease according to cardiovascular-kidney-metabolic syndrome stages by social determinants of health among females and males, with stages 0-2 as reference**

| **CKM stage** | **Social determinants of health** | **Females** | | **Males** | | ***P* for sex interaction** |
| --- | --- | --- | --- | --- | --- | --- |
|  |  | Hazard ratio^a^ | *P* values | Hazard ratio^a^ | *P* values |  |
| **Categories** | **Education** |  |  |  |  | 0.098 |
| Stages 0-2 | High school graduate or higher | 1.00 (Reference) |  | 1.00 (Reference) |  |  |
| Stages 0-2 | Less than high school | 1.04 (0.53-2.07) | 0.890 | 1.57 (0.97-2.52) | 0.064 |  |
| Stages 3-4 | High school graduate or higher | 5.41 (2.93-10.01) | <0.001 | 3.35 (2.11-5.31) | <0.001 |  |
| Stages 3-4 | Less than high school | 8.61 (4.27-17.35) | <0.001 | 4.13 (2.43-6.99) | <0.001 |  |
| **Categories** | **Marital status** |  |  |  |  | 0.137 |
| Stages 0-2 | Married or living with a partner | 1.00 (Reference) |  | 1.00 (Reference) |  |  |
| Stages 0-2 | Not married nor living with a partner | 1.41 (0.78-2.55) | 0.253 | 1.73 (1.09-2.76) | 0.021 |  |
| Stages 3-4 | Married or living with a partner | 6.01 (2.93-12.30) | <0.001 | 3.32 (2.14-5.15) | <0.001 |  |
| Stages 3-4 | Not married nor living with a partner | 9.36 (4.88-17.95) | <0.001 | 4.75 (2.54-8.89) | <0.001 |  |
| **Categories** | **Family income-to-poverty ratio** |  |  |  |  | 0.002 |
| Stages 0-2 | ≥300% | 1.00 (Reference) |  | 1.00 (Reference) |  |  |
| Stages 0-2 | <300% | 3.97 (1.73-9.11) | 0.001 | 1.35 (0.84-2.16) | 0.207 |  |
| Stages 3-4 | ≥300% | 11.54 (3.94-33.85) | <0.001 | 3.37 (1.80-6.33) | <0.001 |  |
| Stages 3-4 | <300% | 21.89 (8.44-56.77) | <0.001 | 3.95 (2.21-7.07) | <0.001 |  |
| **Categories** | **Food security** |  |  |  |  | 0.088 |
| Stages 0-2 | Full security | 1.00 (Reference) |  | 1.00 (Reference) |  |  |
| Stages 0-2 | Marginal, low, or very low security | 1.56 (0.78-3.11) | 0.203 | 1.87 (1.11-3.17) | 0.019 |  |
| Stages 3-4 | Full security | 7.43 (4.02-13.73) | <0.001 | 3.75 (2.40-5.87) | <0.001 |  |
| Stages 3-4 | Marginal, low, or very low security | 7.64 (3.58-16.31) | <0.001 | 3.65 (2.09-6.37) | <0.001 |  |
| **Categories** | **Type of health insurance** |  |  |  |  | 0.049 |
| Stages 0-2 | Private | 1.00 (Reference) |  | 1.00 (Reference) |  |  |
| Stages 0-2 | Government or none | 2.05 (1.01-4.16) | 0.047 | 1.62 (1.02-2.58) | 0.043 |  |
| Stages 3-4 | Private | 12.56 (6.21-25.37) | <0.001 | 3.57 (2.10-6.05) | <0.001 |  |
| Stages 3-4 | Government or none | 8.04 (3.43-18.84) | <0.001 | 4.36 (2.61-7.29) | <0.001 |  |
| **Categories** | **Employment status** |  |  |  |  | 0.185 |
| Stages 0-2 | Employed, student, or retired | 1.00 (Reference) |  | 1.00 (Reference) |  |  |
| Stages 0-2 | Unemployed | 1.15 (0.58-2.30) | 0.685 | 1.46 (0.94-2.28) | 0.095 |  |
| Stages 3-4 | Employed, student, or retired | 5.67 (3.18-10.10) | <0.001 | 3.13 (1.96-5.01) | <0.001 |  |
| Stages 3-4 | Unemployed | 8.34 (3.99-17.43) | <0.001 | 4.43 (2.54-7.73) | <0.001 |  |
| **Categories** | **Home ownership** |  |  |  |  | 0.049 |
| Stages 0-2 | Own home | 1.00 (Reference) |  | 1.00 (Reference) |  |  |
| Stages 0-2 | Rent home or other arrangement | 1.33 (0.65-2.71) | 0.434 | 1.30 (0.79-2.12) | 0.297 |  |
| Stages 3-4 | Own home | 6.63 (3.61-12.16) | <0.001 | 3.40 (2.16-5.35) | <0.001 |  |
| Stages 3-4 | Rent home or other arrangement | 7.98 (3.79-16.8) | <0.001 | 3.22 (1.88-5.51) | <0.001 |  |
| **Categories** | **Regular health-care access** |  |  |  |  | 0.008 |
| Stages 0-2 | At least one regular health-care facility | 1.00 (Reference) |  | 1.00 (Reference) |  |  |
| Stages 0-2 | None or emergency room | 0.92 (0.30-2.77) | 0.878 | 0.74 (0.40-1.37) | 0.340 |  |
| Stages 3-4 | At least one regular health-care facility | 6.50 (3.85-10.98) | <0.001 | 3.17 (2.12-4.73) | <0.001 |  |
| Stages 3-4 | None or emergency room | 3.72 (0.76-18.16) | 0.104 | 1.47 (0.51-4.22) | 0.476 |  |
| **Categories** | **Cumulative unfavorable SDOHs** |  |  |  |  | 0.016 |
| Stages 0-2 | <2 | 1.00 (Reference) |  | 1.00 (Reference) |  |  |
| Stages 0-2 | ≥2 | 2.95 (1.74-5.01) | <0.001 | 2.65 (1.77-3.97) | <0.001 |  |
| Stages 3-4 | <2 | 3.88 (1.57-9.58) | 0.004 | 4.11 (2.37-7.11) | <0.001 |  |
| Stages 3-4 | ≥2 | 18.0 (9.78-33.3) | <0.001 | 8.11 (4.87-13.5) | <0.001 |  |

Abbreviation: SDOHs, social determinants of health.

^a^ All estimates accounted for complex survey designs. Models were adjusted for baseline age, race/ethnicity, education, marital status, family income-to-poverty ratio, food security, type of health insurance, employment status, home ownership, regular health-care access, alcohol consumption, smoking status, physical activity, and medical history of cancer at baseline if applicable.

**eTable 17. Adjusted hazard ratios for risk of premature mortality from cardiovascular disease according to cardiovascular-kidney-metabolic syndrome stages by social determinants of health among females and males, with stages 3-4 as reference**

| **CKM stage** | **Social determinants of health** | **Females** | | **Males** | | ***P* for sex interaction** |
| --- | --- | --- | --- | --- | --- | --- |
|  |  | Hazard ratio^a^ | *P* values | Hazard ratio^a^ | *P* values |  |
| **Categories** | **Education** |  |  |  |  | 0.098 |
| Stages 0-2 | High school graduate or higher | 0.18 (0.10-0.34) | <0.001 | 0.30 (0.19-0.47) | <0.001 |  |
| Stages 0-2 | Less than high school | 0.19 (0.09-0.40) | <0.001 | 0.47 (0.27-0.82) | 0.009 |  |
| Stages 3-4 | High school graduate or higher | 1.00 (Reference) |  | 1.00 (Reference) |  |  |
| Stages 3-4 | Less than high school | 1.59 (0.83-3.06) | 0.163 | 1.23 (0.73-2.06) | 0.427 |  |
| **Categories** | **Marital status** |  |  |  |  | 0.137 |
| Stages 0-2 | Married or living with a partner | 0.17 (0.08-0.34) | <0.001 | 0.30 (0.19-0.47) | <0.001 |  |
| Stages 0-2 | Not married nor living with a partner | 0.23 (0.11-0.51) | <0.001 | 0.52 (0.32-0.86) | 0.012 |  |
| Stages 3-4 | Married or living with a partner | 1.00 (Reference) |  | 1.00 (Reference) |  |  |
| Stages 3-4 | Not married nor living with a partner | 1.56 (0.75-3.23) | 0.231 | 1.43 (0.82-2.49) | 0.205 |  |
| **Categories** | **Family income-to-poverty ratio** |  |  |  |  | 0.002 |
| Stages 0-2 | ≥300% | 0.09 (0.03-0.25) | <0.001 | 0.30 (0.16-0.56) | <0.001 |  |
| Stages 0-2 | <300% | 0.34 (0.14-0.87) | 0.024 | 0.40 (0.23-0.70) | 0.002 |  |
| Stages 3-4 | ≥300% | 1.00 (Reference) |  | 1.00 (Reference) |  |  |
| Stages 3-4 | <300% | 1.90 (0.67-5.40) | 0.228 | 1.17 (0.67-2.05) | 0.578 |  |
| **Categories** | **Food security** |  |  |  |  | 0.088 |
| Stages 0-2 | Full security | 0.13 (0.07-0.25) | <0.001 | 0.27 (0.17-0.42) | <0.001 |  |
| Stages 0-2 | Marginal, low, or very low security | 0.21 (0.09-0.46) | <0.001 | 0.50 (0.28-0.90) | 0.022 |  |
| Stages 3-4 | Full security | 1.00 (Reference) |  | 1.00 (Reference) |  |  |
| Stages 3-4 | Marginal, low, or very low security | 1.03 (0.48-2.22) | 0.945 | 0.97 (0.56-1.68) | 0.920 |  |
| **Categories** | **Type of health insurance** |  |  |  |  | 0.049 |
| Stages 0-2 | Private | 0.08 (0.04-0.16) | <0.001 | 0.28 (0.17-0.48) | <0.001 |  |
| Stages 0-2 | Government or none | 0.16 (0.08-0.32) | <0.001 | 0.45 (0.26-0.80) | 0.006 |  |
| Stages 3-4 | Private | 1.00 (Reference) |  | 1.00 (Reference) |  |  |
| Stages 3-4 | Government or none | 0.64 (0.31-1.34) | 0.235 | 1.22 (0.72-2.09) | 0.458 |  |
| **Categories** | **Employment status** |  |  |  |  | 0.185 |
| Stages 0-2 | Employed, student, or retired | 0.18 (0.10-0.31) | <0.001 | 0.32 (0.20-0.51) | <0.001 |  |
| Stages 0-2 | Unemployed | 0.20 (0.09-0.44) | <0.001 | 0.47 (0.28-0.78) | 0.004 |  |
| Stages 3-4 | Employed, student, or retired | 1.00 (Reference) |  | 1.00 (Reference) |  |  |
| Stages 3-4 | Unemployed | 1.47 (0.72-3.03) | 0.292 | 1.41 (0.87-2.31) | 0.165 |  |
| **Categories** | **Home ownership** |  |  |  |  | 0.049 |
| Stages 0-2 | Own home | 0.15 (0.08-0.28) | <0.001 | 0.29 (0.19-0.46) | <0.001 |  |
| Stages 0-2 | Rent home or other arrangement | 0.20 (0.10-0.39) | <0.001 | 0.38 (0.22-0.67) | 0.001 |  |
| Stages 3-4 | Own home | 1.00 (Reference) |  | 1.00 (Reference) |  |  |
| Stages 3-4 | Rent home or other arrangement | 1.20 (0.63-2.32) | 0.576 | 0.95 (0.56-1.59) | 0.838 |  |
| **Categories** | **Regular health-care access** |  |  |  |  | 0.008 |
| Stages 0-2 | At least one regular health-care facility | 0.15 (0.09-0.26) | <0.001 | 0.32 (0.21-0.47) | <0.001 |  |
| Stages 0-2 | None or emergency room | 0.14 (0.05-0.40) | <0.001 | 0.23 (0.12-0.47) | <0.001 |  |
| Stages 3-4 | At least one regular health-care facility | 1.00 (Reference) |  | 1.00 (Reference) |  |  |
| Stages 3-4 | None or emergency room | 0.57 (0.12-2.72) | 0.479 | 0.46 (0.16-1.32) | 0.148 |  |
| **Categories** | **Cumulative unfavorable SDOHs** |  |  |  |  | 0.016 |
| Stages 0-2 | <2 | 0.26 (0.10-0.64) | 0.004 | 0.24 (0.14-0.42) | <0.001 |  |
| Stages 0-2 | ≥2 | 0.76 (0.31-1.86) | 0.548 | 0.64 (0.38-1.09) | 0.100 |  |
| Stages 3-4 | <2 | 1.00 (Reference) |  | 1.00 (Reference) |  |  |
| Stages 3-4 | ≥2 | 4.65 (1.77-12.22) | 0.002 | 1.97 (1.20-3.26) | 0.008 |  |

Abbreviation: SDOHs, social determinants of health.

^a^ All estimates accounted for complex survey designs. Models were adjusted for baseline age, race/ethnicity, education, marital status, family income-to-poverty ratio, food security, type of health insurance, employment status, home ownership, regular health-care access, alcohol consumption, smoking status, physical activity, and medical history of cancer at baseline if applicable.

**eTable 18. Adjusted hazard ratios for risk of premature mortality from cardiovascular disease according to cardiovascular-kidney-metabolic syndrome stages by social determinants of health across age groups, with stages 0-2 as reference**

| **CKM stage** | **Social determinants of health** | **Middle-aged** | | **Older** | | ***P* for age interaction** |
| --- | --- | --- | --- | --- | --- | --- |
|  |  | Hazard ratio^a^ | *P* values | Hazard ratio^a^ | *P* values |  |
| **Categories** | **Education** |  |  |  |  | 0.492 |
| Stages 0-2 | High school graduate or higher | 1.00 (Reference) |  | 1.00 (Reference) |  |  |
| Stages 0-2 | Less than high school | 0.97 (0.61-1.53) | 0.886 | 2.61 (1.36-4.98) | 0.004 |  |
| Stages 3-4 | High school graduate or higher | 4.76 (2.91-7.78) | <0.001 | 4.18 (2.47-7.09) | <0.001 |  |
| Stages 3-4 | Less than high school | 8.02 (4.62-13.90) | <0.001 | 5.82 (3.14-10.79) | <0.001 |  |
| **Categories** | **Marital status** |  |  |  |  | 0.313 |
| Stages 0-2 | Married or living with a partner | 1.00 (Reference) |  | 1.00 (Reference) |  |  |
| Stages 0-2 | Not married nor living with a partner | 1.41 (0.84-2.35) | 0.193 | 1.39 (0.80-2.43) | 0.239 |  |
| Stages 3-4 | Married or living with a partner | 5.56 (3.19-9.66) | <0.001 | 3.34 (2.04-5.45) | <0.001 |  |
| Stages 3-4 | Not married nor living with a partner | 8.41 (4.71-15.02) | <0.001 | 4.60 (2.57-8.24) | <0.001 |  |
| **Categories** | **Family income-to-poverty ratio** |  |  |  |  | 0.182 |
| Stages 0-2 | ≥300% | 1.00 (Reference) |  | 1.00 (Reference) |  |  |
| Stages 0-2 | <300% | 1.61 (0.95-2.75) | 0.078 | 1.52 (0.82-2.81) | 0.181 |  |
| Stages 3-4 | ≥300% | 7.33 (3.58-15.00) | <0.001 | 3.23 (1.58-6.62) | 0.002 |  |
| Stages 3-4 | <300% | 8.21 (4.48-15.04) | <0.001 | 5.13 (2.64-9.93) | <0.001 |  |
| **Categories** | **Food security** |  |  |  |  | 0.160 |
| Stages 0-2 | Full security | 1.00 (Reference) |  | 1.00 (Reference) |  |  |
| Stages 0-2 | Marginal, low, or very low security | 1.82 (1.02-3.24) | 0.043 | 1.39 (0.68-2.80) | 0.362 |  |
| Stages 3-4 | Full security | 8.27 (5.16-13.25) | <0.001 | 3.26 (2.05-5.18) | <0.001 |  |
| Stages 3-4 | Marginal, low, or very low security | 5.59 (3.11-10.04) | <0.001 | 4.94 (2.56-9.54) | <0.001 |  |
| **Categories** | **Type of health insurance** |  |  |  |  | 0.026 |
| Stages 0-2 | Private | 1.00 (Reference) |  | 1.00 (Reference) |  |  |
| Stages 0-2 | Government or none | 1.82 (1.07-3.10) | 0.027 | 1.76 (1.02-3.01) | 0.041 |  |
| Stages 3-4 | Private | 6.80 (3.65-12.68) | <0.001 | 4.68 (2.81-7.78) | <0.001 |  |
| Stages 3-4 | Government or none | 9.28 (4.67-18.43) | <0.001 | 4.00 (2.30-6.98) | <0.001 |  |
| **Categories** | **Employment status** |  |  |  |  | 0.034 |
| Stages 0-2 | Employed, student, or retired | 1.00 (Reference) |  | 1.00 (Reference) |  |  |
| Stages 0-2 | Unemployed | 1.25 (0.84-1.86) | 0.261 | 0.99 (0.49-2.01) | 0.974 |  |
| Stages 3-4 | Employed, student, or retired | 5.25 (2.92-9.44) | <0.001 | 3.39 (2.15-5.34) | <0.001 |  |
| Stages 3-4 | Unemployed | 7.88 (4.64-13.36) | <0.001 | 3.05 (1.63-5.71) | 0.001 |  |
| **Categories** | **Home ownership** |  |  |  |  | 0.054 |
| Stages 0-2 | Own home | 1.00 (Reference) |  | 1.00 (Reference) |  |  |
| Stages 0-2 | Rent home or other arrangement | 1.31 (0.81-2.12) | 0.273 | 1.72 (0.84-3.51) | 0.139 |  |
| Stages 3-4 | Own home | 5.53 (3.05-10.03) | <0.001 | 4.02 (2.64-6.14) | <0.001 |  |
| Stages 3-4 | Rent home or other arrangement | 7.86 (4.67-13.25) | <0.001 | 2.86 (1.46-5.60) | 0.002 |  |
| **Categories** | **Regular health-care access** |  |  |  |  | 0.179 |
| Stages 0-2 | At least one regular health-care facility | 1.00 (Reference) |  | 1.00 (Reference) |  |  |
| Stages 0-2 | None or emergency room | 0.87 (0.46-1.63) | 0.663 | 1.08 (0.48-2.44) | 0.857 |  |
| Stages 3-4 | At least one regular health-care facility | 6.12 (4.08-9.17) | <0.001 | 3.36 (2.24-5.05) | <0.001 |  |
| Stages 3-4 | None or emergency room | 1.95 (0.65-5.82) | 0.230 | 2.63 (0.75-9.29) | 0.131 |  |
| **Categories** | **Cumulative unfavorable SDOHs** |  |  |  |  | 0.086 |
| Stages 0-2 | <2 | 1.00 (Reference) |  | 1.00 (Reference) |  |  |
| Stages 0-2 | ≥2 | 2.73 (1.75-4.26) | <0.001 | 2.46 (1.45-4.20) | 0.001 |  |
| Stages 3-4 | <2 | 5.62 (2.51-12.54) | <0.001 | 3.81 (2.16-6.71) | <0.001 |  |
| Stages 3-4 | ≥2 | 16.22 (9.74-27.02) | <0.001 | 7.79 (4.50-13.47) | <0.001 |  |

Abbreviation: SDOHs, social determinants of health.

^a^ All estimates accounted for complex survey designs. Models were adjusted for sex, race/ethnicity, education, marital status, family income-to-poverty ratio, food security, type of health insurance, employment status, home ownership, regular health-care access, alcohol consumption, smoking status, physical activity, and medical history of cancer at baseline if applicable.

**eTable 19. Adjusted hazard ratios for risk of premature mortality from cardiovascular disease according to cardiovascular-kidney-metabolic syndrome stages by social determinants of health across age groups, with stages 3-4 as reference**

| **CKM stage** | **Social determinants of health** | **Middle-aged** | | **Older** | | ***P* for age interaction** |
| --- | --- | --- | --- | --- | --- | --- |
|  |  | Hazard ratio^a^ | *P* values | Hazard ratio^a^ | *P* values |  |
| **Categories** | **Education** |  |  |  |  | 0.492 |
| Stages 0-2 | High school graduate or higher | 0.21 (0.13-0.34) | <0.001 | 0.24 (0.14-0.41) | <0.001 |  |
| Stages 0-2 | Less than high school | 0.20 (0.11-0.39) | <0.001 | 0.62 (0.37-1.05) | 0.075 |  |
| Stages 3-4 | High school graduate or higher | 1.00 (Reference) |  | 1.00 (Reference) |  |  |
| Stages 3-4 | Less than high school | 1.68 (0.89-3.20) | 0.111 | 1.39 (0.87-2.21) | 0.162 |  |
| **Categories** | **Marital status** |  |  |  |  | 0.313 |
| Stages 0-2 | Married or living with a partner | 0.18 (0.10-0.31) | <0.001 | 0.30 (0.18-0.49) | <0.001 |  |
| Stages 0-2 | Not married nor living with a partner | 0.25 (0.14-0.46) | <0.001 | 0.42 (0.25-0.71) | 0.001 |  |
| Stages 3-4 | Married or living with a partner | 1.00 (Reference) |  | 1.00 (Reference) |  |  |
| Stages 3-4 | Not married nor living with a partner | 1.51 (0.78-2.94) | 0.220 | 1.38 (0.86-2.21) | 0.180 |  |
| **Categories** | **Family income-to-poverty ratio** |  |  |  |  | 0.182 |
| Stages 0-2 | ≥300% | 0.14 (0.07-0.28) | <0.001 | 0.31 (0.15-0.63) | 0.002 |  |
| Stages 0-2 | <300% | 0.22 (0.10-0.49) | <0.001 | 0.47 (0.24-0.93) | 0.030 |  |
| Stages 3-4 | ≥300% | 1.00 (Reference) |  | 1.00 (Reference) |  |  |
| Stages 3-4 | <300% | 1.12 (0.47-2.65) | 0.796 | 1.59 (0.82-3.07) | 0.170 |  |
| **Categories** | **Food security** |  |  |  |  | 0.160 |
| Stages 0-2 | Full security | 0.12 (0.08-0.19) | <0.001 | 0.31 (0.19-0.49) | <0.001 |  |
| Stages 0-2 | Marginal, low, or very low security | 0.22 (0.13-0.38) | <0.001 | 0.43 (0.23-0.78) | 0.006 |  |
| Stages 3-4 | Full security | 1.00 (Reference) |  | 1.00 (Reference) |  |  |
| Stages 3-4 | Marginal, low, or very low security | 0.68 (0.36-1.27) | 0.220 | 1.52 (0.90-2.55) | 0.113 |  |
| **Categories** | **Type of health insurance** |  |  |  |  | 0.026 |
| Stages 0-2 | Private | 0.15 (0.08-0.27) | <0.001 | 0.21 (0.13-0.36) | <0.001 |  |
| Stages 0-2 | Government or none | 0.27 (0.14-0.50) | <0.001 | 0.38 (0.22-0.65) | 0.001 |  |
| Stages 3-4 | Private | 1.00 (Reference) |  | 1.00 (Reference) |  |  |
| Stages 3-4 | Government or none | 1.36 (0.63-2.96) | 0.430 | 0.86 (0.50-1.47) | 0.571 |  |
| **Categories** | **Employment status** |  |  |  |  | 0.034 |
| Stages 0-2 | Employed, student, or retired | 0.19 (0.11-0.34) | <0.001 | 0.30 (0.19-0.47) | <0.001 |  |
| Stages 0-2 | Unemployed | 0.24 (0.13-0.45) | <0.001 | 0.29 (0.15-0.59) | 0.001 |  |
| Stages 3-4 | Employed, student, or retired | 1.00 (Reference) |  | 1.00 (Reference) |  |  |
| Stages 3-4 | Unemployed | 1.50 (0.73-3.10) | 0.272 | 0.90 (0.50-1.62) | 0.728 |  |
| **Categories** | **Home ownership** |  |  |  |  | 0.054 |
| Stages 0-2 | Own home | 0.18 (0.10-0.33) | <0.001 | 0.25 (0.16-0.38) | <0.001 |  |
| Stages 0-2 | Rent home or other arrangement | 0.24 (0.14-0.39) | <0.001 | 0.43 (0.21-0.87) | 0.020 |  |
| Stages 3-4 | Own home | 1.00 (Reference) |  | 1.00 (Reference) |  |  |
| Stages 3-4 | Rent home or other arrangement | 1.42 (0.81-2.49) | 0.217 | 0.71 (0.39-1.30) | 0.265 |  |
| **Categories** | **Regular health-care access** |  |  |  |  | 0.179 |
| Stages 0-2 | At least one regular health-care facility | 0.16 (0.11-0.24) | <0.001 | 0.30 (0.20-0.45) | <0.001 |  |
| Stages 0-2 | None or emergency room | 0.14 (0.07-0.28) | <0.001 | 0.32 (0.14-0.71) | 0.006 |  |
| Stages 3-4 | At least one regular health-care facility | 1.00 (Reference) |  | 1.00 (Reference) |  |  |
| Stages 3-4 | None or emergency room | 0.32 (0.10-0.97) | 0.044 | 0.78 (0.23-2.63) | 0.690 |  |
| **Categories** | **Cumulative unfavorable SDOHs** |  |  |  |  | 0.086 |
| Stages 0-2 | <2 | 0.18 (0.08-0.40) | <0.001 | 0.26 (0.15-0.46) | <0.001 |  |
| Stages 0-2 | ≥2 | 0.49 (0.21-1.10) | 0.082 | 0.65 (0.37-1.12) | 0.117 |  |
| Stages 3-4 | <2 | 1.00 (Reference) |  | 1.00 (Reference) |  |  |
| Stages 3-4 | ≥2 | 2.89 (1.27-6.55) | 0.011 | 2.04 (1.23-3.40) | 0.006 |  |

Abbreviation: SDOHs, social determinants of health.

^a^ All estimates accounted for complex survey designs. Models were adjusted for sex, race/ethnicity, education, marital status, family income-to-poverty ratio, food security, type of health insurance, employment status, home ownership, regular health-care access, alcohol consumption, smoking status, physical activity, and medical history of cancer at baseline if applicable.

**eReferences**

[1] Kidney Disease: Improving Global Outcomes CKDWG. KDIGO 2024 Clinical Practice Guideline for the Evaluation and Management of Chronic Kidney Disease. Kidney Int. 2024;105:S117-S314.

[2] Khan SS, Matsushita K, Sang Y, Ballew SH, Grams ME, Surapaneni A, et al. Development and validation of the American Heart Association Predicting Risk of Cardiovascular Disease EVENTs (PREVENT) equations. Circulation. 2023;149:430–49.

[3] Inker LA, Eneanya ND, Coresh J, Tighiouart H, Wang D, Sang YY, et al. New Creatinine- and Cystatin C-Based Equations to Estimate GFR without Race. New Engl J Med. 2021;385:1737-49.

[4] Bundy J, Mills K, He H, Laveist TA, Ferdinand KC, Chen J, et al. Social determinants of health and premature death among adults in the USA from 1999 to 2018: a national cohort study. Lancet Public Health. 2023;8:E422-E31.

[5] Va P, Dodd KW, Zhao L, Thompson-Paul AM, Mercado CI, Terry AL, et al. Evaluation of measurement error in 24-hour dietary recall for assessing sodium and potassium intake among US adults—National Health and Nutrition Examination Survey (NHANES), 2014. Am J Clin Nutr. 2019;109:1672-82.

[6] Murakami K, Livingstone MB. Prevalence and characteristics of misreporting of energy intake in US adults: NHANES 2003-2012. Br J Nutr. 2015;114:1294-303.
